# Neighborhood environment and socioeconomic inequalities in cancer admissions: a prospective study using UK Biobank and linked hospital records

**DOI:** 10.1007/s10552-022-01626-2

**Published:** 2022-09-18

**Authors:** Kate E. Mason, Neil Pearce, Steven Cummins

**Affiliations:** 1 Department of Public Health Policy and Systems, University of Liverpool, Liverpool, UK; 2 Department of Non-Communicable Disease Epidemiology, London School of Hygiene and Tropical Medicine, London, UK; 3 Department of Medical Statistics, London School of Hygiene and Tropical Medicine, London, UK; 4 Department of Public Health, Environments and Society, London School of Hygiene and Tropical Medicine, London, UK

**Keywords:** Cancer, Epidemiology, Environment, Urban design, Green space, Hospital admissions, Socioeconomic factors

## Abstract

**Purpose:**

Neighborhood environments may influence cancer risk. Average population effect estimates might mask differential effects by socioeconomic position. Improving neighborhood environments could inadvertently widen health inequalities if important differences are overlooked.

**Methods:**

Using linked records of hospital admissions in UK Biobank, we assessed associations between admission with a primary diagnosis of cancer (any/breast/colorectal), and exposure to neighborhood greenspace, physical activity facilities, and takeaway food stores, and whether household income and area deprivation modify these associations. We used adjusted Cox proportional hazards models, and estimated relative excess risks due to interaction (RERI) to assess effect modification.

**Results:**

Associations between neighborhood exposures and cancer-related hospitalizations were weak to null overall, but with some evidence of effect modification. Most notably, more greenspace near home was associated with 16% lower hazard of cancer-related hospital admission in deprived areas (95% CI 2–29%). This was further pronounced for people in low-income households in deprived areas, and for breast cancer.

**Conclusion:**

In deprived neighborhoods, increasing the amount of greenspace may help reduce cancer-related hospitalizations. Examining effect modification by multiple socioeconomic indicators can yield greater insight into how social and environmental factors interact to influence cancer incidence. This may help avoid perpetuating cancer inequalities when designing neighborhood environment interventions.

**Supplementary Information:**

The online version contains supplementary material available at 10.1007/s10552-022-01626-2.

## Background

Residential neighborhood environments have the potential to influence cancer risk by promoting or hindering physical activity and healthy diets and exacerbating or mitigating chronic stress. Some environmental exposures, such as greenspace, may also act as a buffer against physical environmental hazards, such as air pollution, and boost immune function, potentially offering further protection against cancer [1, 2]. The unequal distribution of these neighborhood exposures by key socioeconomic factors may also contribute to inequalities in cancer risk and mortality.

The evidence base for neighborhood effects on cancer is dominated by studies of neighborhood socioeconomic status and ethnicity, with few studies examining characteristics of the neighborhood built environment [3]. This contrasts with other chronic conditions such as cardiovascular disease, outcomes such as obesity, and various health-related behaviors, all of which have been extensively studied in relation to neighborhood built environment exposures, albeit with inconsistent findings [4–7]. Given the overlap in more proximal risk factors for cancer and other chronic diseases (e.g., diet, physical activity, obesity, stress) neighborhood features might also affect cancer risk. Specific features of the neighborhood built environment that have been hypothesized to influence cancer include green spaces such as public parks and private gardens, the retail food environment (including proximity or density of healthy and unhealthy food stores), and accessibility of formal recreation facilities for physical activity (such as public swimming pools, gyms, sports fields) among others [3]. The potential for food and formal physical activity environments to influence health outcomes such as cancer via diet and physical activity is straightforward. Greenspace may exert influence through a wider range of mechanisms, including facilitating recreational physical activity or functional physical activity such as gardening or active travel, but also via other pathways relating to the regulation of stress hormones, improved immune function through exposure to diverse microorganisms, and reduced exposure to air pollution [8], all of which may influence risk of cancer.

Cross-sectional studies predominate in neighborhood health effects research but a recent shift toward more longitudinal studies—in part facilitated by increasing ability to link hospital records and routine data to population-based cohorts with geographical data on neighborhood environments—is creating rich opportunities to examine whether neighborhood environmental exposures are associated with objectively recorded, prospective outcomes, thus helping to better elucidate the true causal relationships and mechanisms involved, e.g., [9–11]. However, recent reviews reveal a paucity of longitudinal research on built environments and cancer risk, and substantial heterogeneity among the studies that do exist, in terms of cancer sites, exposure and outcome measures, and populations [3, 12]. The limited evidence base from these studies is inconsistent, not only between but also within studies. One recent study of cancer and greenspace in France, for example, suggests a potentially protective role against breast cancer for some greenspace measures, but increased risk of other cancers linked to alternative greenspace measures [13].

Causal neighborhood effects on cancer are likely to be small, and part of a broader swathe of environmental, social and structural drivers of health behaviors and outcomes, each contributing incrementally to the complex physical and social environments that influence our ability to make healthy lifestyle choices and mitigate the stresses of modern life. An important aspect of better understanding these relationships is the possibility that they are not uniform across the population, but that some population subgroups and geographical areas are more sensitive to their neighborhood environment than others. Important effects concentrated in particular population subgroups or particular places may be masked by average, population-wide estimates.

Socioeconomic differences may be one source of such effect heterogeneity. For various health outcomes, studies exist that suggest heterogeneous neighborhood health effects according to individual socioeconomic conditions [14, 15] or neighborhood deprivation [16, 17]. These may arise if preferences for particular neighborhood resources vary according to individual socioeconomic conditions, regardless of the physical availability of neighborhood resources, e.g., if low-income households tend to make more use of fast-food/takeaway stores, or if access to gyms and leisure centers is restricted to people with high incomes because of membership fees. Or differences may arise if the quality or accessibility of resources that are present in an area is unevenly distributed spatially, according to area-level deprivation (rather than individual/household socioeconomic conditions), e.g., if more deprived areas have poorer quality public greenspace. On the other hand, if, for example, greenspace promotes health without an attendant increase in financial costs to the individual, then access to local greenspace may offset inequitable access to formal physical activity facilities, and therefore have a larger effect in deprived areas or for low-income households. Regardless of the direction of any such heterogeneity of effect, it remains a poorly understood aspect of the relationship between the neighborhood environment and health. If differential benefits or harms of neighborhood characteristics are observed by measures of individual socioeconomic conditions, such as household income, or by neighborhood deprivation, then any efforts to improve population health by improving neighborhood built environments (e.g., increasing availability of physical activity facilities or reducing the number of fast-food outlets near residential areas) may widen health inequalities if they are blind to socially differential impacts [18].

In this paper, we use baseline UK Biobank data on neighborhood exposures to physical activity facilities, greenspace and fast-food stores, linked to records of subsequent hospital admissions up to January 2016, to examine (1) the relative hazard of being admitted to hospital with a primary diagnosis of cancer, according to exposure to each of the neighborhood characteristics, and (2) whether there is evidence of effect modification of those associations by household income and/or area deprivation.

## Methods

### Data and study design

#### UK Biobank

We used data from UK Biobank (project 17380﻿) the scientific rationale, study design, and survey methods for which have been described elsewhere [19]. More than half a million individuals were recruited to visit one of 22 UK Biobank assessment centers across the UK between 2006 and 2010, where they completed a touchscreen questionnaire, took part in a face-to-face nurse-led interview and had a series of physical measurements taken. All individuals aged 40–69 years living within a 25-mile radius of an assessment center and listed on National Health Service (NHS) patient registers were invited to participate in the study. The age range was chosen by UK Biobank as an important period for the development of many chronic diseases.

**Fig. 1:**
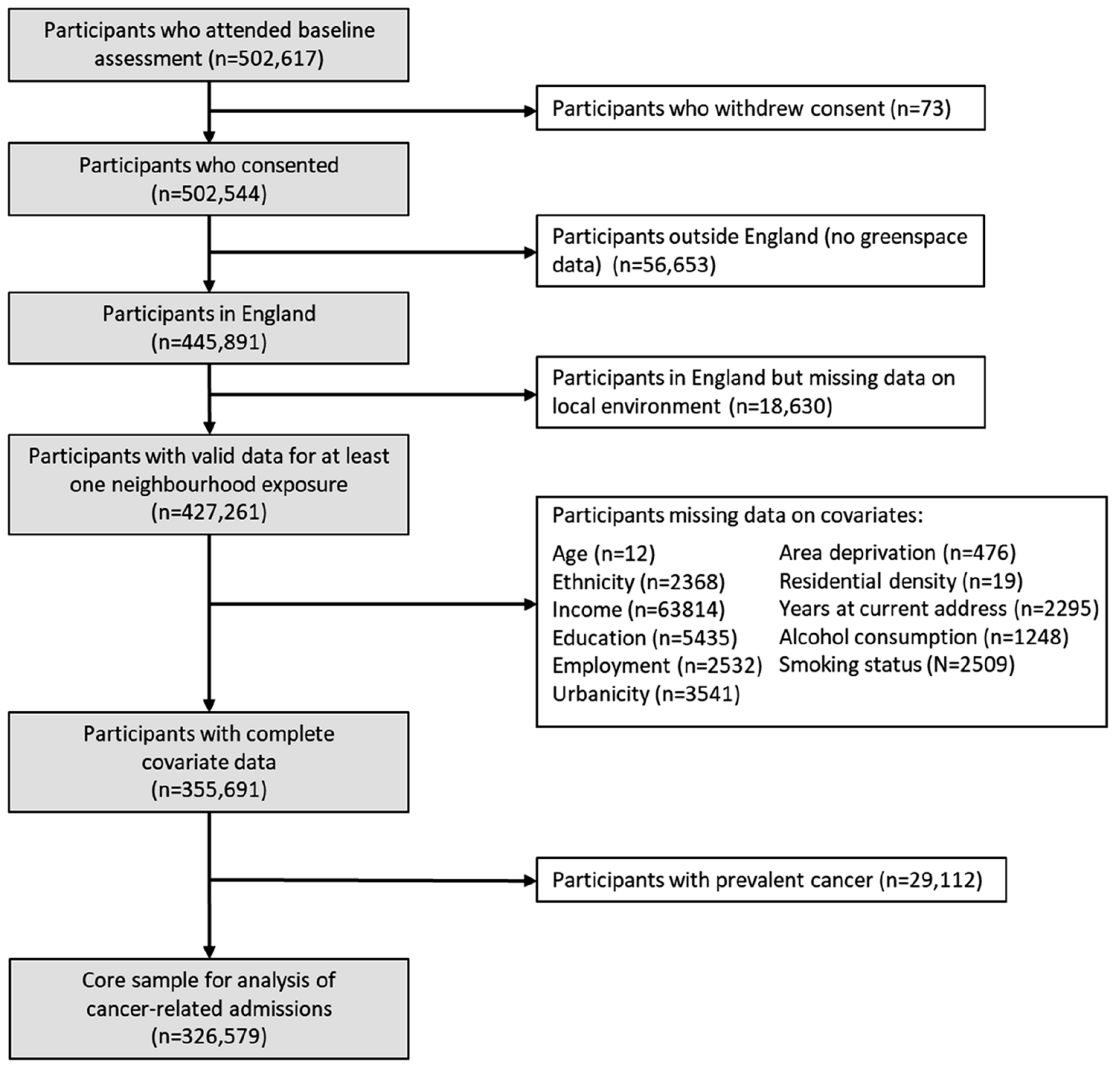
UK Biobank sample for analyses

Of the 502,617 participants in UK Biobank for whom some data were available, 355,691 remained after excluding withdrawals and restricting the sample to individuals who lived in England (greenspace data were only available for England) and had data for at least one measure of the neighborhood environment and complete data on covariates. Of these, we excluded 29,112 individuals who reported a previous cancer diagnosis at baseline (in the baseline interview where a trained nurse collected data on past and current medical conditions), leaving a possible *n* = 326,579 for analysis (Fig. 1). The final analytic sample sizes varied according to availability of the neighborhood variable under examination. The maximum follow-up time after baseline assessment was 9.8 years but varied according to the date of an individual’s recruitment to the study.

#### Neighborhood environment data

Linked to UK Biobank is a high-resolution spatial database of a range of objectively measured characteristics of the physical environment surrounding each participant’s exact residential address, known as the UK Biobank Urban Morphometric Platform (UKBUMP). Environmental data in UKBUMP were derived, using automated processes, from multiple pre-existing sources roughly contemporaneous with the individual baseline assessment [20]. In this study, we use physical activity facility and food outlet data based on land use classifications contained in the UKBUMP and derived from the UK Ordnance Survey AddressBase Premium database [20]. Over time, as researchers work with UK Biobank, new linked data are being made available to the research community, including additional environmental measures of greenspace [21] that we have used here in addition to measures from the original UKBUMP. More details are provided below.

#### Linked hospital admissions data

Ongoing prospective linkage of the cohort to administrative health records is a key feature of the UK Biobank resource. At the time of analysis, linked Hospital Episode Statistics were available up to January 2016. These contain information on hospital admissions coded using the International Statistical Classification of Diseases and Related Health Problems, 10th Revision (ICD‐10). We used these data to identify incident admissions to hospital for cancer. Any subsequent admissions were ignored.

### Measures

#### Outcomes

Outcomes were any hospital admission for which the primary diagnosis is recorded as cancer (ICD-10 codes C00-C97). In a set of secondary analyses, we examine breast cancer (C50) and colorectal cancers (C18-20) specifically, as these have some of the strongest links to physical activity [22] and, to a lesser extent, diet [23]— two potential mediators of the neighborhood effects being examined.

#### Neighborhood exposures

Three measures of the neighborhood built environment were examined. To account for non-normal distributions and the potential for non-linear relationships with the outcome, and to facilitate a categorical approach to the analysis of effect modification within a time-to-event setting [24], each exposure was split into four categories. The exposures we examined were:

1. Availability of physical activity facilities: number of formal physical activity facilities within a one-kilometer street network distance of each participant’s home address, categorized as 0, 1, 2-3, or 4 or more. Physical activity facilities were defined at address level as any land use classified in the Commercial-Leisure subcategory of the UK Ordnance Survey AddressBase Premium database. This subcategory comprises a range of indoor and outdoor facilities designed for sporting and leisure activities, such as gyms, swimming pools, and playing fields (see supplementary material for details). Informal physical activity facilities, such as public parks, are not included, except where covered by the above classification, e.g., playing fields. A 1 km buffer has been used in numerous other studies; it equates to about a 10–15-min walk and has been reported to be roughly the area that people perceive to be their neighborhood [25].
2. Fast-food proximity: street network distance in meters from participants’ home address to the nearest outlet classified as a ‘hot/cold fast-food outlet/takeaway’ in the UK Ordnance Survey AddressBase Premium database, categorized as < 500 m, 500–999 m, 1000–1999 m, 2000 m + .
3. Greenspace: percentage of 300 m Euclidean buffer around home address classified as ‘greenspace’ or ‘domestic garden’ in the Generalized Land Use Database (GLUD). 'Greenspace' in the GLUD includes all public or private vegetated areas larger than 5 m^2^ in area, with the exception of domestic gardens, which are classified separately. We combined ‘greenspace’ and ‘gardens’ into a single measure, consistent with previous research using the GLUD to examine relationships with health [9]. A 300 m buffer was chosen to capture greenspace in the immediate vicinity of a person’s home. There is some evidence that 300 m is a distance from home beyond which the use of green spaces quickly declines [26, 27], and it has been proposed in the UK as a benchmark for greenspace provision [28]. Greenspace was grouped into quartiles.

The measures underlying exposures (1) and (2) were derived by Sarkar et al. in the UKBUMP from OS AddressBase Premium 2012 [20] at the end of the UK Biobank baseline data collection period, while (3) was derived by Wheeler et al. from the Generalized Land Use Database 2005 [21] (see source documentation for further detail). We restricted the analyses to people residing in England, because the greenspace data for exposure (3) were not available for UK Biobank participants in Wales and Scotland.

#### Potential effect modifiers

We examined whether the association between each neighborhood exposure and cancer admissions was modified by binary indicators for annual, pre-tax household income (< £31,000 or ≥ £31,000) and area deprivation (most deprived 40% of UK census output areas vs least deprived 60%). Income data were collected by UK Biobank in five broad bands, and we used the cutpoint of £31,000 when constructing our binary indicator, as this splits the sample roughly in half at a value approximating the household income of a two-person household of 55–59 year olds where one person earns the 2010 median income for that age and the other earns half the median [29]. Area deprivation category was based on the 2001 Townsend index score of the census output area in which each individual’s home postcode was located, with the binary indicator constructed using publicly available quintile cutpoints for the Townsend index. Census output areas are small statistical units comprising, in the large majority of cases, between 110 and 139 households. Using the Townsend index ensured our deprivation measure was based on census data collected prior to baseline. We dichotomized the potential effect modifiers into two similarly sized groups rather than comparing a more extreme end of the distribution to the majority, to maximize statistical power to detect interactions. When testing for effect modification, household income and area deprivation were combined with each primary exposure into a categorical variable capturing all combinations of levels of the exposure and potential modifier, with a single reference category (see below for details). We also examined the combined modifying role of income and deprivation. Area-based and individual indicators of social disadvantage have been shown to contribute to health outcomes independently of one another, providing a rationale for examining them both in parallel and in combination [30].

#### Potential confounders

Based on information from previous studies, we identified potential confounders of the primary associations as age (years), sex (binary), ethnicity (White/non-White), educational qualifications (college or university degree; post compulsory secondary education (A/AS levels); secondary education (O levels) or below/other qualifications), employment status (paid work; retired; unable to work; unemployed; or other), urban/non-urban, UK Biobank assessment center, and neighborhood residential density (count of residential dwellings within a 1-km street network buffer of home address, log transformed). Annual household income (< £18,000, £18,000–30,999, £31,000–51,999, ≥ £52,000) and area deprivation (Townsend score) were also included as possible confounders in any models when not being tested as a potential effect modifier. We also adjusted models for smoking status (current/previous/never) and alcohol intake frequency (less than/at least three times per week) as these are important risk factors for the outcome and may be correlated with neighborhood, and number of years living at current (baseline) address to at least partially condition on pre-baseline exposure to neighborhood environment, which could act as a confounder. Confounder data were ascertained during the baseline assessment (or in the case of residential density, through linkage to the UKBUMP). We do not adjust for diet or physical activity, although they are risk factors for the outcome. Dietary data are only available for a selected subset (about half) of the analytical sample, so including it in our models would severely reduce our sample size and the study’s statistical power, as well as potentially biasing our results. Furthermore, in the food environment models, it is also a hypothesized mediator. Physical activity is omitted because it is a likely mediator of the associations with physical activity environment and, potentially, greenspace.

### Statistical analysis

Baseline characteristics were summarized by the mean (and standard deviation) or median (and interquartile range) for continuous variables and number (and percent) for categorical variables. We then examined associations between neighborhood exposure and incident hospital admission due to cancer following baseline assessment and up to 31 January 2016, using multivariable Cox proportional hazard models, with adjustment for potential confounders and censoring for death. Results are expressed as hazard ratios (HRs) and 95% confidence intervals (95% CI). The proportional hazards assumption was tested by visual inspection of adjusted log–log plots (Supplementary Fig. S3). The reference categories for each neighborhood exposure are the hypothetically least health-promoting (lowest availability of physical activity facilities, shortest distance to nearest fast-food store, least greenspace).

We examined whether the primary associations were modified by area deprivation and household income. In line with STROBE recommendations [31] and using the method described by Li and Chambless [32] and VanderWeele [24], the relative excess risk due to interaction (RERI) was estimated to assess effect modification on the additive scale. When dealing with binary and time-to-event outcomes, the decision to examine effect modification on either the multiplicative or the additive scale has implications for interpretation. The additive scale provides important information about the potential public health consequences of intervening on the exposure, in different strata of the effect modifier. This is not information that can usually be estimated directly from an examination of effect modification on the multiplicative scale [24]. The RERI is estimated by first estimating the HR for each combination of the exposure and potential modifier values relative to a single reference category, in this case the least hypothetically health-promoting level of the respective neighborhood variable (no physical activity facilities; < 500 m from nearest fast-food store; or quartile with least greenspace), and either low income (< £31,000) or more deprived area (home address located in a census output area in the most deprived 40% of all UK areas). In other words, the reference category in each analysis is the group expected to have the highest baseline risk of the outcome. Then, taking the HRs from this model for the least and most exposed groups, the RERI is estimated as:

$${\text{RERI }} = {\text{ HR}}_{{{11}}} {-}{\text{ HR}}_{{{1}0}} {-}{\text{ HR}}_{{0{1}}} + { 1}$$

For the model assessing effect modification of physical activity facility availability by household income, for example, HR_11_ represents the HR (relative to the reference category) for people in high-income households (at least £31,000 per year) and who have four or more physical activity facilities within 1000 m of home; HR_10_ represents the HR for people in low-income households with four or more physical activity facilities near home; and HR_01_ is the HR for people in high-income households with no physical activity facilities near home.

For the models of the other neighborhood exposures, and models of effect modification by area deprivation, subscript one represents those most exposed to the potentially health-promoting neighborhood exposure and less deprived areas, respectively. As such, a RERI value greater than zero—which implies a positive departure from additivity—suggests that in this case any estimated protective effect of the neighborhood variable among people in low-income households or in more deprived areas is greater than the estimated protective effect among people from high-income households or less deprived areas. In contrast, a RERI < 0 suggests any protective effect of the neighborhood variable is greater in the high-income/less deprived group. By contrasting the two extreme categories of exposure, we assume this is the most relevant contrast.

As recommended by Knol and VanderWeele [33], we also report estimates of effect modification on the multiplicative scale (HR_11_/HR_10_×HR_01_,), and income- and deprivation stratum-specific HRs comparing the groups least and most exposed to each neighborhood exposure. Finally, we also separately estimated HRs for each stratum of income and area deprivation in combination, and for each stratum, we conducted a test for trend by fitting each exposure as a continuous variable.

All analyses were conducted using Stata v14.2 (StataCorp LP, College Station, TX, USA). While the primary analyses for all cancer and colorectal cancer were adjusted for sex, we also repeated these analyses separately for males and females. Analysis of breast cancer admissions was restricted to participants recorded as female.

#### Sensitivity analyses

The spatial data used in the creation of the UKBUMP to ascertain the neighborhood food and physical activity exposures were recorded in 2012, just after the baseline data collection period [20]. While it is assumed that neighborhood exposure will be sufficiently constant over this period, we check this assumption by conducting a sensitivity analysis in which follow-up is restricted to the period from 2012 onwards for all participants, rather than from the baseline assessment date (which could be as early as 2006). Separately, we checked whether results were sensitive to additional adjustment for baseline hypertension, BMI, and medications for hypertension or cholesterol—predictors of the outcome that we did not include in the main analysis because of ambiguity regarding temporal precedence i.e., they may be on the causal pathways from neighborhood environment to cancer if neighborhood exposure predates them.

## Results

### Descriptive

Table 1 summarizes the characteristics of the study participants at baseline assessment. The sample has a mean age of 56 years at baseline and was predominantly of White ethnicity and urban dwelling. Reflecting the age of the sample, just over half were educated to no higher than compulsory secondary education level, and six in every ten were employed at baseline. Participants were evenly distributed across income categories, with roughly half living in households with an annual gross income below £31,000, while 29% lived in the more deprived 40% of areas in the UK.

**Table 1:** Descriptive characteristics of sample (*n* = 326,579)

Variable	Participants admitted for cancer (*n* = 13,935)	Participants not admitted for cancer (*n* = 312,644)
Female (n, %)	6,649 (47.7%)	162,014 (51.8%)
Age (years) (mean, SD)	60.0 (6.8)	55.8 (8.1)
Non-White ethnicity (*n*, %)	461 (3.3%)	16,389 (5.2%)
Urban (*n*, %)	11,882 (85.3%)	267,904 (85.7%)
Education (*n*, %)		
College or University degree	4,191 (30.1%)	107,940 (34.5%)
Post-compulsory secondary education (A/AS levels or equivalent)	1,360 (9.8%)	36,691 (11.7%)
Secondary education (O levels or equivalent) or below/other qualification	8,384 (60.2%)	168,013 (53.7%)
Employment status (n, %)		
Paid work	6,559 (47.1%)	195,137 (62.4%)
Retired	6,382 (45.8%)	92,596 (29.6%)
Unable to work	416 (3.0%)	8,532 (2.7%)
Unemployed	180 (1.3%)	4,960 (1.6%)
Other	398 (2.9%)	11,419 (3.7%)
Residential density (residential sites per 1000 m buffer) (median, IQR)	1890 (1096–3055)	1922 (1110–3133)
Years at current address (median, IQR)	20 (9–29)	15 (7–25)
Area deprivation (mean Townsend score, SD)	− 1.4 (3.0)	− 1.4 (3.0)
Area deprivation (n, % in two most deprived quintiles of the UK)	4,043 (29.0%)	91,775 (29.4%)
Household income (n, %)		
< £18,000	4,001 (28.7%)	68,428 (21.9%)
£18,000–30,999	4,125 (29.6%)	78,729 (25.2%)
£31,000–51,999	3,251 (23.3%)	82,594 (26.4%)
£52,000 or more	2,558 (18.4%)	82,893 (26.5%)
Smoking status (n, %)		
Current	1,824 (13.1%)	31,965 (10.2%)
Previous	5,658 (40.6%)	108,064 (34.6%)
Never	6,453 (46.3%)	172,615 (55.2%)
Frequency of alcohol consumption (*n*, % ≥ 3 times per week)	6,523 (46.8%)	141,315 (45.2%)

Column total may not add to 100% due to rounding

The mean follow-up time for participants was 6.8 years. Over the follow-up period, 13,935 individuals (4.27%) were admitted to hospital with cancer (Table 2). Proportionally, there were more hospital admissions among people from low-income households, whereas admissions were similar across levels of area deprivation.

**Table 2:** Hospital admissions by household income and area deprivation

	N	Number of cancer admissions (%)
Total	326,579	13,935 (4.3)
Household income (annual pre-tax)*		
< £31,000	155,283	8,126 (5.2)
£31,000 or more	171,296	5,809 (3.4)
Area deprivation**		
More deprived	95,818	4,043 (4.2)
Less deprived	230,761	9,892 (4.3)

*Self-reported average total household income before tax. **More deprived' refers to people living in areas in the top two most deprived quintiles of the UK, based on the Townsend index

#### Associations between neighborhood characteristics and admissions for all cancer types

**Fig. 2:**
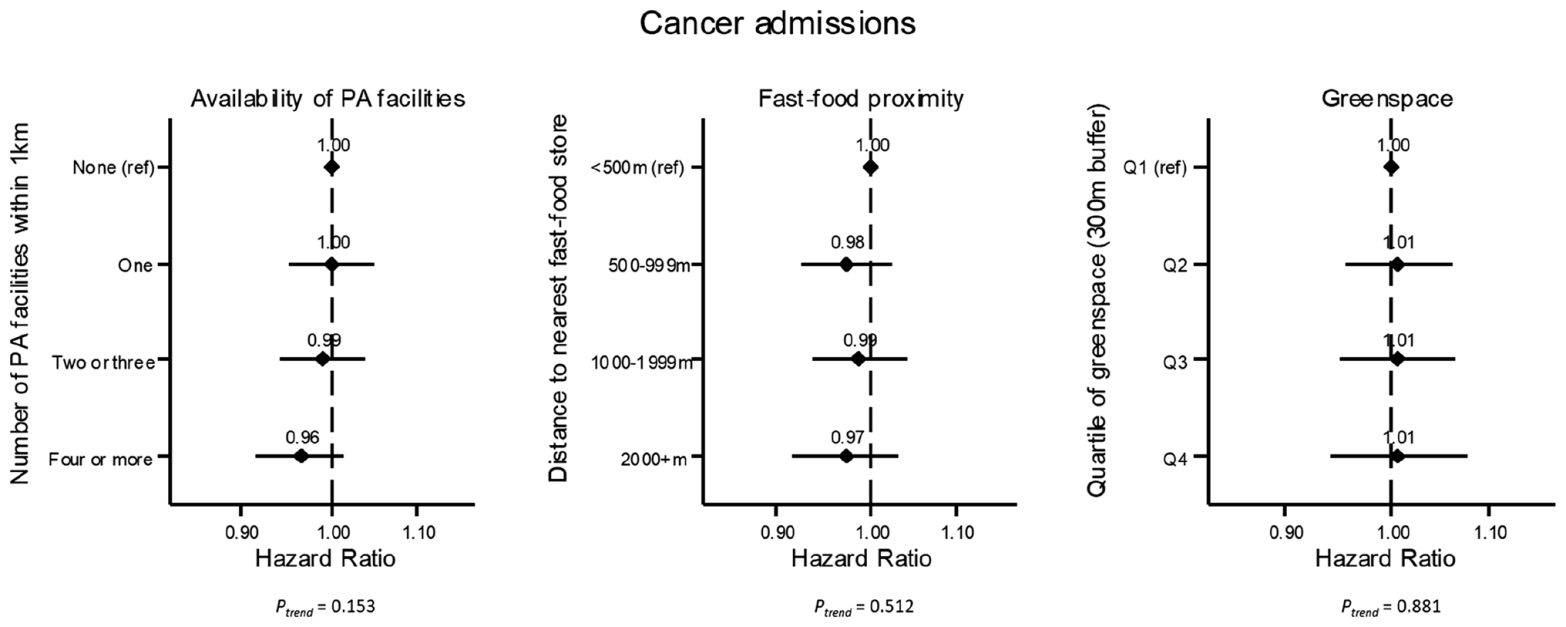
Adjusted hazard ratios for hospital admission due to cancer, by availability of formal physical activity (PA) facilities, proximity to nearest fast-food/takeaway store, and neighborhood greenspace. Models are adjusted for age, sex, ethnicity, education, household income, employment status, urban/non-urban, assessment area, residential density, smoking status, alcohol intake, and number of years living at home address. For plots from sex-stratified models, see Supplementary Material Fig. S1

Figure 2 summarizes the hazard ratios for hospital admissions due to cancer associated with each of the three neighborhood environment measures, across the sample as a whole. While 95% CIs for all associations include 1, there was some indication of a slightly lower hazard of cancer-related hospital admission among those people with at least four physical activity facilities within one kilometer of their home, compared to people with no nearby formal physical activity facilities (HR = 0.96; 95% CI 0.91–1.01), but no evidence that one, two or three facilities offers a benefit compared to the reference. For fast-food proximity and neighborhood greenspace, we observed no association with risk of cancer-related admission when averaging across the study population as a whole.

#### Modification of associations between neighborhood characteristics and cancer-related hospital admissions, by income and area deprivation

The association between physical activity facilities and all cancer admissions does not appear to be modified by income or by area deprivation. Stratum-specific HRs were similar across socioeconomic groups, and RERIs were close to zero for both potential effect modifiers (Table 3).

**Table 3:** Modification of the association between built environment variables and hospital admissions due to cancer, by household income and area deprivation

Cancer-related admissions	Annual household income*		Area deprivation**	
	< £31,000	At least £31,000	More deprived	Less deprived
	HR (95% CI)	HR (95% CI)	HR (95% CI)	HR (95% CI)
Number of physical activity facilities				
None (ref)	1.00	0.96 (0.90, 1.02) *p* = 0.209	1.00	1.02 (0.94, 1.11) *p* = 0.605
One	0.98 (0.92, 1.05) *p* = 0.588	0.98 (0.91, 1.06) *p* = 0.608	1.07 (0.97, 1.19) *p* = 0.175	1.00 (0.92, 1.09) *p* = 0.961
2–3	1.00 (0.94, 1.06) *p* = 0.918	0.94 (0.87, 1.00) *p* = 0.066	1.06 (0.96, 1.16) *p* = 0.249	0.99 (0.91, 1.08) *p* = 0.844
4 or more	0.99 (0.93, 1.05) *p* = 0.676	0.89 (0.83, 0.96) *p* = 0.001	1.01 (0.92, 1.11) *p* = 0.846	0.98 (0.90, 1.06) *p* = 0.581
Stratum-specific HRs (4 + facilities vs. 0)	0.97 (0.91, 1.04) *p* = 0.395	0.95 (0.87, 1.03) *p* = 0.184	1.02 (0.92, 1.13) *p* = 0.673	0.95 (0.89, 1.01) *p* = 0.110
Relative excess risk due to interaction (RERI) (additive scale)	− 0.06 (− 0.15, 0.03) *p* = 0.197		− 0.05 (− 0.16, 0.05) *p* = 0.320	
Ratio measure of effect modification on multiplicative scale^	0.94 (0.85, 1.02) *p* = 0.184		0.95 (0.85, 1.05) *p* = 0.347	
Fast-food proximity				
Closer than 500 m (ref)	1.00	0.89 (0.82, 0.97) *p* = 0.006	1.00	0.92 (0.85, 0.99) *p* = 0.029
500–999 m	0.94 (0.88, 1.00) *p* = 0.053	0.93 (0.86, 1.00) *p* = 0.039	0.90 (0.83, 0.97) *p* = 0.009	0.94 (0.88, 1.01) *p* = 0.083
1000–1999 m	0.98 (0.92, 1.04) *p* = 0.472	0.90 (0.84, 0.97) P = 0.008	0.99 (0.91, 1.08) *p* = 0.843	0.92 (0.85, 0.98) *p* = 0.017
At least 2000 m	0.94 (0.88, 1.01) *p* = 0.098	0.91 (0.84, 0.99) P = 0.021	0.93 (0.84, 1.03) *p* = 0.165	0.92 (0.85, 0.99) *p* = 0.024
Stratum-specific HRs (≥ 2000 m vs < 500 m)	0.97 (0.90, 1.05) *p* = 0.451	0.97 (0.89, 1.07) P = 0.591	0.93 (0.83, 1.04) *p* = 0.187	1.00 (0.93, 1.07) *p* = 0.966
Relative excess risk due to interaction (RERI) (additive scale)	0.08 (− 0.01, 0.18) *p* = 0.088		0.07 (-0.04, 0.18) *p* = 0.218	
Ratio measure of effect modification on multiplicative scale^	1.09 (0.98, 1.20) *p* = 0.095		1.08 (0.95, 1.21) *p* = 0.214	
Greenspace				
Q1 (least greenspace) (ref)	1.00	0.86 (0.80, 0.93) *p* = 0.000	1.00	0.93 (0.87, 1.01) *p* = 0.070
Q2	0.98 (0.92, 1.05) *p* = 0.595	0.91 (0.84, 0.98) *p* = 0.012	0.99 (0.92, 1.07) *p* = 0.764	0.95 (0.89, 1.01) *p* = 0.129
Q3	0.96 (0.90, 1.03) *p* = 0.249	0.94 (0.87, 1.01) *p* = 0.112	1.00 (0.91, 1.10) *p* = 0.976	0.95 (0.89, 1.01) *p* = 0.094
Q4 (most greenspace)	0.95 (0.87, 1.02) *p* = 0.166	0.96 (0.88, 1.04) *p* = 0.287	0.86 (0.75, 0.98) *p* = 0.024	0.96 (0.89, 1.04) *p* = 0.325
Stratum-specific HRs (Q4 vs Q1)	0.97 (0.89, 1.06) *p* = 0.490	1.05 (0.95, 1.17) *p* = 0.336	0.84 (0.71, 0.98) *p* = 0.027	1.04 (0.96, 1.13) *p* = 0.332
Relative excess risk due to interaction (RERI) (additive scale)	0.15 (0.06, 0.24) *p* = 0.001		0.17 (0.04, 0.30) *p* = 0.008	
Ratio measure of effect modification on multiplicative scale^	1.17 (1.06, 1.29) *p* = 0.002		1.20 (1.03, 1.37) *p* = 0.012	

Models are adjusted for age, sex, ethnicity, education, employment status, urban/non-urban, assessment area, residential density, smoking status, alcohol intake, and number of years living at home address

*Self-reported average total household income before tax. **More deprived' refers to people living in areas in the top two most deprived quintiles of the UK, based on the Townsend index. Q = quartile. ^ HR_11_/HR_10_×HR_01_

In contrast, there was some evidence of effect modification by socioeconomic factors for the associations between the other neighborhood exposures and hospitalization for cancer. The most marked evidence was for a modifying effect of area deprivation on the association between greenspace and cancer-related admissions. In that case, the positive departure from additivity indicated by the RERI of 0.17 suggests the reductions in admissions associated with increased exposure to neighborhood greenspace may be greater in more deprived areas (Table 3). In more deprived areas, the stratum-specific HRs estimate a 16% lower hazard of cancer-related hospitalization among those in the greenest quartile compared with those from the least green quartile (HR = 0.84; 95% CI 0.71–0.98), while no association was observed among people living in less deprived areas. A similar pattern was observed for fast-food proximity and cancer-related admissions, albeit with a smaller and non-significant departure from additivity (RERI = 0.07) and a smaller estimated reduction in hazard among the more deprived areas (HR = 0.93; 95% CI 0.83–1.04). For household income, although the RERIs for both fast-food proximity and greenspace did indicate some departure from additivity, the stratum-specific HRs suggested there was no meaningful association between these neighborhood exposures and cancer-related admissions in either income group (Table 3).

Combining area deprivation and household income, a beneficial association of having greater exposure to greenspace within 300 m of home was observed among low-income households in deprived areas, where the hazard of cancer-related hospital admission was 24% lower among people living in the greenest quartile than among people living in the least green quartile (HR = 0.76, 95% CI 0.63–0.92, Table 4). The test for trend was not significant in this stratum and intermediate quartiles showed no significant difference from the least green quartile, but all HRs were less than one.

**Table 4:** Association between neighborhood characteristics and cancer-related hospital admissions, stratified by household income and area deprivation in combination

Cancer-related admissions	Combined household income and area deprivation			
	Less than £31,000 & more deprived	At least £31,000 & more deprived	Less than £31,000 & less deprived	At least £31,000 & less deprived
	HR (95% CI)	HR (95% CI)	HR (95% CI)	HR (95% CI)
Number of physical activity facilities				
None	1.00 (ref)	1.00 (ref)	1.00 (ref)	1.00 (ref)
One	1.05 (0.93, 1.18) *p* = 0.452	1.14 (0.93, 1.40)* p* = 0.212	0.95 (0.88, 1.03)* p* = 0.203	1.02 (0.94, 1.10)* p* = 0.694
2–3	1.07 (0.95, 1.19) *p* = 0.256	1.05 (0.87, 1.28)* p* = 0.598	0.96 (0.89, 1.03)* p* = 0.258	0.98 (0.90, 1.07)* p* = 0.656
4 or more	1.05 (0.93, 1.18) *p* = 0.434	0.98 (0.81, 1.19)* p* = 0.850	0.94 (0.86, 1.02)* p* = 0.152	0.96 (0.87, 1.05)* p* = 0.324
P_*trend*_	0.448	0.436	0.151	0.295
Fast-food proximity				
Closer than 500 m	1.00 (ref)	1.00 (ref)	1.00 (ref)	1.00 (ref)
500–999 m	0.89 (0.81, 0.98)* p* = 0.015	0.93 (0.80, 1.08)* p* = 0.347	1.00 (0.92, 1.10)* p* = 0.945	1.06 (0.96, 1.18)* p* = 0.254
1000–1999 m	0.97 (0.87, 1.08)* p* = 0.569	1.01 (0.86, 1.20)* p* = 0.899	1.03 (0.94, 1.12)* p* = 0.572	0.98 (0.88, 1.09)* p* = 0.733
At least 2000 m	0.88 (0.77, 1.01)* p* = 0.063	1.06 (0.85, 1.32)* p* = 0.607	1.02 (0.93, 1.13)* p* = 0.655	0.98 (0.88, 1.10)* p* = 0.743
P_*trend*_	0.166	0.627	0.552	0.264
Greenspace				
Q1 (least greenspace)	1.00 (ref)	1.00 (ref)	1.00 (ref)	1.00 (ref)
Q2	0.98 (0.90, 1.08)* p* = 0.720	0.97 (0.83, 1.13)* p* = 0.654	1.02 (0.93, 1.13)* p* = 0.612	1.03 (0.93, 1.15)* p* = 0.566
Q3	0.96 (0.85, 1.08)* p* = 0.456	1.03 (0.84, 1.26)* p* = 0.768	1.01 (0.92, 1.12)* p* = 0.803	1.05 (0.94, 1.18)* p* = 0.357
Q4 (most greenspace)	0.76 (0.63, 0.92)* p* = 0.005	1.05 (0.78, 1.40)* p* = 0.752	1.04 (0.93, 1.16)* p* = 0.543	1.08 (0.95, 1.22)* p* = 0.233
P_*trend*_	0.039	0.776	0.665	0.218

Models are adjusted for age, sex, ethnicity, education, employment status, urban/non-urban, assessment area, residential density, smoking status, alcohol intake, and number of years living at home address

*Self-reported average total household income before tax. **'More deprived' refers to people living in areas in the top two most deprived quintiles of the UK, based on the Townsend index

Q = quartile

People from low-income households in deprived areas were also the group where living at least 2 km from a fast-food store had the strongest association with cancer-related admissions (HR = 0.88, 95% CI 0.77–1.01), but there was no clear trend of decreasing hazard with decreasing proximity (Table 4).

No income/deprivation combined subgroup appeared to experience a cancer-related benefit of having more physical activity facilities near home, although there was some evidence that those in low-income households in less deprived areas had a somewhat lower hazard (6%) if they had at least four physical activity facilities with a kilometer of home, compared with no facilities (HR = 0.94, 95% CI 0.86–1.02, Table 4).

#### Sex differences

For the relationships between all three neighborhood exposures and admissions for any cancer, the findings were generally consistent for females and males (Supplementary Fig. S1 and Tables S1–S3).

#### Secondary outcomes: breast and colorectal cancer

When we explored whether the results for cancer hospitalizations were driven by either of the two cancers most strongly linked to some of the plausible pathways by which neighborhood characteristics might influence cancer risk (namely breast and colorectal cancer), we found that the evidence of effect modification by area deprivation of the association between greenspace and cancer admission was magnified for breast cancer (RERI = 0.32, Supplementary Table S4) and the same was true for effect modification by household income (RERI = 0.31). In deprived areas, the hazard of being admitted to hospital with a primary diagnosis of breast cancer was 31% lower among females with the greatest exposure to neighborhood greenspace, compared with females who had the least greenspace near home (HR = 0.69, 95% CI 0.47–0.99, Supplementary Table S4). No such association was observed for females living in less deprived areas, and no association was observed between greenspace and breast cancer admissions for the whole sample. For females from lower-income households who also lived in deprived areas, risk of a breast cancer-related admission was 39% lower among those with the greatest exposure to greenspace, compared with those who had the least exposure (HR = 0.61, 95% CI 0.38–0.97, Supplementary Table S5). Within this combined deprivation/income stratum, the relationship did not appear to be linear; rather only those living in the greenest areas showed lower hazard of cancer admission.

For formal physical activity facilities, no overall association was observed with either cancer subtype (Supplementary Fig. S2), just as was the case for all cancers combined. However, lower risk of admission for colorectal cancer appeared to be associated with greater availability of physical activity facilities among people living in less deprived areas, and in particular among people from lower-income households within less deprived areas (Supplementary Tables S6 and S7). Contrasting this, there was some indication of effect modification by income for breast cancer admissions, such that females from higher income households were less likely to be hospitalized with breast cancer if they have at least four physical activity facilities near home (Supplementary Table S4).

For fast-food proximity, neither cancer type showed an association with this neighborhood exposure and there was limited evidence of any effect modification by either income or deprivation (Supplementary Fig. S2 and Table S6).

### Sensitivity analyses

In general, restricting follow-up to the period from 2012 onwards for all participants, rather than from the baseline assessment date, reduced precision around point estimates, but made minimal difference to the overall direction and magnitude of most coefficients and RERI estimates (Supplementary Tables S9 and S10). Results were also robust to adjustment for additional risk factors for cancer (Supplementary Tables S11 and S12).

## Discussion

Across this very large sample of mid-aged adults in the UK, we examined the relationship between three characteristics of the neighborhood built environment and hospital admissions due to cancer, over almost 10 years of follow-up. We examined whether these associations were modified by area deprivation and household income, with the aim of identifying which neighborhood characteristics might be suitable candidates for interventions to improve health without widening existing health inequalities.

We observed very little evidence that any of the three neighborhood exposures were associated with overall hospitalizations due to cancer. However, investigation of effect modification by household income and area deprivation uncovered interesting patterns that may help to illuminate important elements of the links between the neighborhood built environment and health. The largely null overall associations appeared to mask potentially important variations in the strength and magnitude of some of those associations by sex, individual socioeconomic conditions, and area deprivation.

For neighborhood greenspace and cancer-related hospital admissions—and particularly for women admitted for breast cancer—we found evidence of effect modification by area deprivation, suggesting any protective influence of greenspace against cancer may be greater in more deprived areas than in less deprived areas. This finding is consistent with some other studies that have previously found relationships between greenspace and health to be stronger in more deprived communities [1], including in the UK [34, 35]. In contrast, there did not appear to be any association between formal physical activity facilities and cancer within any income or area deprivation subgroup, and conflicting patterns of effect modification by income for breast and colorectal cancer specifically.

One pathway through which urban greenspace is hypothesized to influence health is via physical activity [36]. However, the fact we observed no association between physical activity facilities and cancer, including in deprived areas, combined with other, as yet unpublished findings in which we found no relationship between greenspace and CVD, suggests that greenspace might influence health generally through pathways unrelated to physical activity. This contrasts, to some extent, with a 2016 study in the US in which physical activity was estimated to explained a small proportion (2%) of an observed association between greenspace and cancer mortality [11], but is consistent with recent studies from Spain and the US that reported associations between urban greenspace and breast cancer [37] and lethal prostate cancer [38] that were unlikely to be mediated by physical activity. While formal physical activity facilities are unlikely to influence health via pathways other than through physical activity itself, there is emerging evidence that greenspace may influence health via multiple pathways, including reduced exposure to environmental stressors such noise, heat and air pollution, mental wellbeing, and immune function [8, 36], as well as physical activity. Several studies have concluded that greenspace-health relationships, if causal, are mediated by pathways other than physical activity, most notably psychosocial ones [11, 39, 40]. One mechanism by which greenspace is thought to influence health is the regulation of cortisol secretion [41, 42]. While short-term cortisol secretion is a protective physiological response to stress, chronically elevated cortisol levels can cause dysregulation of the body’s glucocorticoid system and has been associated with various health outcomes including cancer [43, 44]. A recent study in a deprived setting in Scotland found that the presence of more greenspace near the home was associated with lower levels of stress across objective cortisol secretion measures and subjective measures of stress, but this relationship did not appear to be mediated by physical activity [41]. Access to greenspace near home may also plausibly mitigate other biological pathways through which chronic psychological stress (more prevalent in deprived populations) influences cancer risk, such as oxidative stress-induced DNA damage and telomere shortening [45, 46]. Similarly, greenspace may mitigate some of the effects on cancer risk of air and noise pollution (also often higher in deprived areas), operating through these and related inflammatory and oxidative stress pathways [47, 48].

For fast-food proximity and cancer, there was only very weak evidence that area deprivation modifies this association, and no evidence of an interaction with income. The measure of fast-food proximity we have used is somewhat problematic, however, and these results may not be reliable. There is likely to be some systematic misclassification, random error, and geographical inconsistency in quality in the proximity measure we have used, due to our reliance on an off-the shelf measure based on local authority data sources collected for non-research purposes. This highlights some of the trade-offs made in the use of big data and administrative data for the purposes of epidemiological research. Further research repeating this England-wide analysis using improved measures of the fast-food environment may clarify this relationship.

An important a priori rationale for examining effect modification by factors such as income and area deprivation, when a study is sufficiently powered to do so, is that it is plausible that some groups of people will be more sensitive to their neighborhood environment than others, and that some may be almost completely insensitive for various reasons. Population-wide, average effect estimates smooth out these differences and potentially lead to erroneous conclusions about the importance of neighborhood environments for some people in some places. Indeed, in this study, we found very little evidence of association between these neighborhood exposures and cancer across the study population overall, but stronger evidence for associations were observed within more deprived subgroups. We would only expect small point estimates overall, given the complexity and multitude of causes of cancer, and how distal the outcome is from the exposures, but nonetheless, in some cases, these population-average null findings contradict what we might expect based on previous research. In particular, evidence from food environment research in the UK has been mounting of a detrimental effect of excessive exposure to unhealthy food outlets [15, 49–51]. Limitations of the fast-food proximity measure are described above and are also likely to have led to conservative estimates. Similarly, the greenspace measure may not adequately capture the full extent of relevant greenness of one’s neighborhood, as it does not include smaller parcels of greenspace such as street trees or reflect ‘quality’ of greenspace. Despite the very large sample size, the small main effect estimates for the neighborhood exposures will have limited the statistical power of our study such that we were only able to detect moderately strong interactions.

There are several other limitations of the current study. First, the hospital admissions data only capture inpatient care, so any early detection of cancer that occurs in primary care settings after baseline and is then effectively treated without admission to hospital will not be counted. Such cases are probably more likely to occur in higher income or less deprived subgroups [52], and this may have contributed to lower risk of hospital admission in those groups, potentially distorting the magnitude of effect modification on the additive scale. In future, when primary care records are fully linked to the UK Biobank cohort, it will be possible to examine this potential source of bias. Related to this, if some types of health care have shifted to outpatient settings over the course of the follow-up period, it may result in some dilution of the true association overall and between subgroups. We have not distinguished between elective and emergency admissions, and differences in these may also be socially patterned. Future research could also make use of linked cancer registry data to explore these associations further.

Second, it is unclear what period of follow-up is optimal, given that people will have been exposed to their baseline neighborhood conditions for varying lengths of time depending on how long they have lived at that address, and whether relevant changes had occurred in their neighborhood during that time, and the nature of previous neighborhood exposures. We adjusted our analyses for years living at baseline address to attempt to deal with this, and are reassured by the long average time people have lived at the address we are using (median = 15 years). However, there may be remaining imprecision, and potential bias of estimates in either direction, that we cannot overcome using observational data of this kind. Longer follow-up may prove to be more revealing, and that will become possible in future years, but ideally future work would also account for changes in the built environment over that period. UK Biobank would be made richer by the addition of measurement of neighborhood exposures at one or more post-baseline time points. Our sensitivity analyses using a shorter follow-up period to account for the timing of the exposure ascertainment showed that most point estimates were robust to this specification, but there was a loss of precision presumably driven by the substantial reduction in the number of hospital admissions occurring during the shortened follow-up period (Supplementary Table S9).

Third, we cannot rule out potential selection bias from several sources. The UK Biobank sample is not representative of the wider UK population [53]. On top of this, further selection resulted from our exclusion of 15% of the potential sample due to missing income data. We also cannot exclude the possibility of self-selection into more health-promoting neighborhoods by people more disposed to healthy behaviors. We can, however, by the longitudinal nature of the study and exclusion of people with prevalent disease at baseline, rule out active self-selection prior to baseline into neighborhoods on the basis of prevalent disease (e.g., following a cancer diagnosis earlier in life, deciding to relocate to a neighborhood more supportive of a healthy lifestyle). This means that we likely minimize masking of the true extent of association via this avenue, but may still have some residual positive confounding that could bias estimates away from the null, despite our comprehensive adjustment for observed potential confounders. However, UK Biobank is a residentially very stable sample, and most of our strongest findings were within more deprived subgroups, where financial resources enabling relocation for health purposes are presumably the least. In robustness checks we also confirmed that further model adjustment for baseline hypertension, BMI, and medications for hypertension or cholesterol, made no material difference to our findings. Remaining sources of potential residual confounding we were not able to explore may include access to care. However, confounding by this is likely to be at least partly controlled for by other covariates such as urban/non-urban status and assessment area, and is not likely to have been a major factor in the UK setting where healthcare is free at the point of delivery. Thus, residual confounding cannot be excluded, but is unlikely to be a major source of bias.

In summary, despite no overall association between greenspace and cancer admissions across the mid-aged English population, we did find evidence of effect modification by area deprivation. Living in a neighborhood with a greater percentage of greenspace is associated with lower risk of cancer-related hospitalization among people living in more deprived areas. There is some evidence of the same being true for reduced fast-food proximity and cancer. Greater availability of physical activity facilities close to home is not associated with lower risk of cancer for any of the analyzed groups. Improving deprived neighborhoods by increasing the amount of public and private greenspace and limiting the proximity of fast-food outlets to residential areas may improve health outcomes in the population.

Taken together, these results suggest that improving access to greenspace may have a greater public health impact in more deprived areas, but the pathway(s) by which these benefits might arise require further elucidation and should not be assumed to be restricted to the promotion and facilitation of physical activity. We also show that by examining effect modification by multiple socioeconomic indicators in parallel, potentially important insights can be gained that may be missed when we focus only on a single socioeconomic measure. Understanding the potentially different ways in which different aspects of the socioeconomic conditions of people’s lives influence their relationship with the built environment and its effects on cancer risk may help to avoid intervention-generated inequalities when neighborhood-based built environment interventions are designed.

## Supplementary Information

Below is the link to the electronic supplementary material.

Supplementary file1 (DOCX 677 kb)

## Data Availability

All data used in this study are available to approved researchers on application to UK Biobank https://www.ukbiobank.ac.uk/
