# Supplementary material for: Neighborhood environment and socioeconomic inequalities in cancer admissions: a prospective study using UK Biobank and linked hospital records
Source: Cancer Causes Control. 2022 Sep 18;33(12):1431–44. doi: 10.1007/s10552-022-01626-2 (PMC9630224; doi:10.1007/s10552-022-01626-2)
Supplement: Supplementary file 1 — Supplementary file1 (DOCX 677 kb) [file 10552_2022_1626_MOESM1_ESM.docx]

## SUPPLEMENTARY MATERIAL

**Neighbourhood environment and socioeconomic inequalities in cancer admissions: a prospective study using UK Biobank and linked administrative records**

**Kate E. Mason*, Neil Pearce, Steven Cummins**

*Corresponding Author: [kate.mason@liverpool.ac.uk](mailto:kate.mason@liverpool.ac.uk)

**1. Classification of Formal Physical Activity Facilities**

Formal PA facilities were defined as any land use classified in the Commercial-Leisure subcategory (CL06) of the UK Ordnance Survey AddressBase Premium database (<https://www.ordnancesurvey.co.uk/business-and-government/help-and-support/products/addressbase-premium.html>). The data are contributed by local authorities, and covers municipal and private facilities for all sporting activities. This subcategory comprises any Indoor/Outdoor Leisure/Sporting Activity/Centre not further defined, as well as the following more specific categories of land use:

- Bowls Facility
- Cricket Facility
- Diving / Swimming Facility
- Equestrian Sports Facility
- Football Facility
- Golf Facility
- Activity / Leisure / Sports Centre
- Playing Field
- Racquet Sports Facility
- Rugby Facility
- Recreation Ground
- Skateboarding Facility
- Civilian Firing Facility
- Tenpin Bowling Facility
- Water Sports Facility
- Winter Sports Facility

Full details of the classification scheme and the types of facilities covered can be found via the link above.

**2. Primary analysis stratified by sex**

**Figure S1: Hazard ratios for associations between neighbourhood characteristics and all cancer-related hospital admissions, stratified by sex**

**
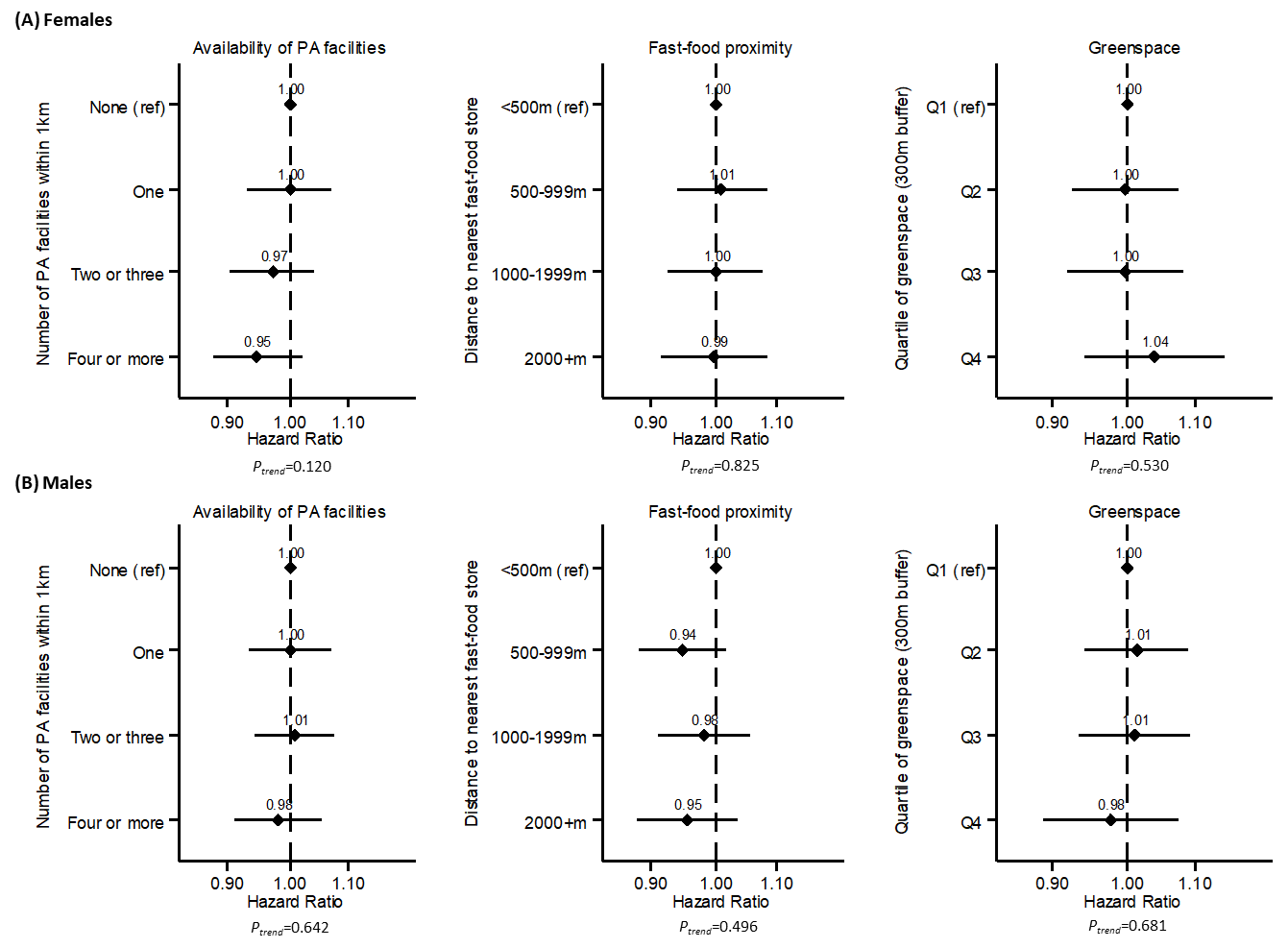
**

Note: Models are adjusted for age, ethnicity, education, household income, employment status, urban/non-urban, assessment area, residential density, smoking status, alcohol intake, and number of years living at home address.

**Table S1. Modification of the association between neighbourhood availability of PA facilities and hospital admissions due to cancer, by household income and area deprivation, stratified by sex**

|  | **Annual household income*** | | | | **Area deprivation**** | | | |
| --- | --- | --- | --- | --- | --- | --- | --- | --- |
|  | **FEMALES** | | **MALES** | | **FEMALES** | | **MALES** | |
|  | **< £31,000** | **At least £31,000** | **< £31,000** | **At least £31,000** | **More deprived** | **Less deprived** | **More deprived** | **Less deprived** |
| **Number of PA facilities** | HR (95% CI); P | HR (95% CI); P | HR (95% CI); P | HR (95% CI); P | HR (95% CI); P | HR (95% CI); P | HR (95% CI); P | HR (95% CI); P |
|  |  |  |  |  |  |  |  |  |
| None | 1.00 (ref) | 0.89 (0.81, 0.98) P=0.018 | 1.00 (ref) | 1.03 (0.94, 1.13) P=0.482 | 1.00 (ref) | 1.08 (0.96, 1.21) P=0.217 | 1.00 (ref) | 0.97 (0.87, 1.09) P=0.602 |
| One | 0.95 (0.87, 1.04) P=0.236 | 0.96 (0.87, 1.07) P=0.501 | 1.02 (0.93, 1.11) P=0.703 | 1.00 (0.91, 1.11) P=0.932 | 1.09 (0.94, 1.26) P=0.251 | 1.05 (0.93, 1.19) P=0.407 | 1.06 (0.92, 1.22) P=0.421 | 0.95 (0.85, 1.07) P=0.415 |
| 2-3 | 0.98 (0.90, 1.07) P=0.668 | 0.85 (0.77, 0.94) P=0.002 | 1.02 (0.93, 1.11) P=0.700 | 1.02 (0.93, 1.13) P=0.652 | 1.13 (0.99, 1.29) P=0.081 | 0.99 (0.87, 1.12) P=0.876 | 1.00 (0.88, 1.13) P=0.951 | 0.99 (0.88, 1.11) P=0.865 |
| 4 or more | 0.96 (0.88, 1.05) P=0.401 | 0.82 (0.74, 0.91) P=0.000 | 1.01 (0.93, 1.11) P=0.742 | 0.96 (0.87, 1.06) P=0.405 | 1.00 (0.87, 1.14) P=0.959 | 1.02 (0.90, 1.16) P=0.758 | 1.02 (0.90, 1.16) P=0.745 | 0.94 (0.83, 1.05) P=0.270 |
|  |  |  |  |  |  |  |  |  |
| Stratum-specific HRs  (4+ facilities vs 0) | 0.94 (0.85, 1.03) P=0.181 | 0.96 (0.85, 1.08) P=0.532 | 1.01 (0.92, 1.11) P=0.864 | 0.94 (0.84, 1.05) P=0.243 | 1.03 (0.89, 1.19) P=0.711 | 0.94 (0.86, 1.02) P=0.152 | 1.02 (0.89, 1.17) P=0.808 | 0.96 (0.88, 1.05) P=0.391 |
|  |  |  |  |  |  |  |  |  |
| Relative excess risk due to interaction (RERI) | -0.03 (-0.15, 0.09)  P=0.584 | | -0.09 (-0.22, 0.04)  P=0.172 | | -0.05 (-0.21, 0.10)  P=0.502 | | -0.06 (-0.20, 0.09)  P=0.454 | |
| Ratio measure of effect modification on multiplicative scale^ | 0.96 (0.83, 1.08) P=0.555 | | 0.91 (0.80, 1.03) P=0.144 | | 0.95 (0.80, 1.10) P=0.526 | | 0.94 (0.81, 1.08) P=0.406 | |

* Self-reported average total household income before tax.

** 'More deprived' refers to people living in areas in the top two most deprived quintiles of the UK, based on the Townsend index.

^ HR_11_ / HR_10_ x HR_01_

Note: Models are adjusted for age, ethnicity, education, employment status, urban/non-urban, assessment area, residential density, smoking status, alcohol intake, and number of years living at home address.

**Table S2. Modification of the association between fast-food proximity and hospital admissions due to cancer, by household income and area deprivation, stratified by sex**

|  | **Annual household income*** | | | | **Area deprivation**** | | | |
| --- | --- | --- | --- | --- | --- | --- | --- | --- |
|  | **FEMALES** | | **MALES** | | **FEMALES** | | **MALES** | |
|  | **< £31,000** | **At least £31,000** | **< £31,000** | **At least £31,000** | **More deprived** | **Less deprived** | **More deprived** | **Less deprived** |
| **Fast-food proximity** | HR (95% CI); P | HR (95% CI); P | HR (95% CI); P | HR (95% CI); P | HR (95% CI); P | HR (95% CI); P | HR (95% CI); P | HR (95% CI); P |
|  |  |  |  |  |  |  |  |  |
| Closer than 500m | 1.00 (ref) | 0.78 (0.69, 0.88) P=0.000 | 1.00 (ref) | 1.00 (0.89, 1.12) P=0.971 | 1.00 (ref) | 0.92 (0.82, 1.03) P=0.138 | 1.00 (ref) | 0.91 (0.82, 1.01) P=0.088 |
| 500-999m | 0.94 (0.86, 1.03) P=0.174 | 0.90 (0.81, 1.00) P=0.050 | 0.94 (0.86, 1.03) P=0.160 | 0.95 (0.86, 1.06) P=0.349 | 0.95 (0.85, 1.06) P=0.368 | 0.97 (0.87, 1.07) P=0.527 | 0.85 (0.77, 0.95) P=0.005 | 0.91 (0.83, 1.00) P=0.058 |
| 1000-1999m | 0.97 (0.88, 1.06) P=0.461 | 0.84 (0.76, 0.94) P=0.002 | 0.99 (0.90, 1.08) P=0.762 | 0.97 (0.87, 1.07) P=0.547 | 0.95 (0.84, 1.08) P=0.452 | 0.95 (0.86, 1.05) P=0.314 | 1.03 (0.92, 1.15) P=0.649 | 0.89 (0.80, 0.98) P=0.015 |
| At least 2000m | 0.93 (0.84, 1.03) P=0.141 | 0.88 (0.79, 0.99) P=0.028 | 0.96 (0.87, 1.06) P=0.394 | 0.95 (0.85, 1.05) P=0.311 | 0.94 (0.81, 1.09) P=0.420 | 0.95 (0.85, 1.06) P=0.320 | 0.92 (0.79, 1.06) P=0.235 | 0.89 (0.80, 0.98) P=0.024 |
|  |  |  |  |  |  |  |  |  |
| Stratum-specific HRs  (≥2000m vs <500m) | 0.95 (0.85, 1.06) P=0.364 | 1.08 (0.93, 1.24) P=0.314 | 0.99 (0.89, 1.10) P=0.847 | 0.90 (0.79, 1.02) P=0.101 | 0.93 (0.79, 1.10) P=0.403 | 1.03 (0.93, 1.15) P=0.521 | 0.91 (0.78, 1.07) P=0.264 | 0.97 (0.88, 1.07) P=0.515 |
|  |  |  |  |  |  |  |  |  |
| Relative excess risk due to interaction (RERI) | 0.17 (0.05, 0.30)  P=0.007 | | -0.01 (-0.15, 0.13)  P=0.886 | | 0.09 (-0.08, 0.25)  P=0.294 | | 0.06 (-0.10, 0.21)  P=0.451 | |
| Ratio measure of effect modification on multiplicative scale^ | 1.22 (1.03, 1.40) P=0.011 | | 0.99 (0.85, 1.13) P=0.898 | | 1.10 (0.90, 1.29) P=0.297 | | 1.06 (0.88, 1.24) P=0.516 | |

* Self-reported average total household income before tax.

** 'More deprived' refers to people living in areas in the top two most deprived quintiles of the UK, based on the Townsend index.

^ HR_11_ / HR_10_ x HR_01_

Note: Models are adjusted for age, ethnicity, education, employment status, urban/non-urban, assessment area, residential density, smoking status, alcohol intake, and number of years living at home address.

**Table S3. Modification of the association between neighbourhood greenspace and hospital admissions due to cancer, by household income and area deprivation, stratified by sex**

|  | **Annual household income** | | | | **Area deprivation** | | | |
| --- | --- | --- | --- | --- | --- | --- | --- | --- |
|  | **FEMALES** | | **MALES** | | **FEMALES** | | **MALES** | |
|  | **< £31,000** | **At least £31,000** | **< £31,000** | **At least £31,000** | **More deprived** | **Less deprived** | **More deprived** | **Less deprived** |
| **Greenspace** | HR (95% CI); P | HR (95% CI); P | HR (95% CI); P | HR (95% CI); P | HR (95% CI); P | HR (95% CI); P | HR (95% CI); P | HR (95% CI); P |
|  |  |  |  |  |  |  |  |  |
| Q1 (least greenspace) | 1.00 (ref) | 0.79 (0.71, 0.88) P=0.000 | 1.00 (ref) | 0.93 (0.84, 1.04) P=0.216 | 1.00 (ref) | 0.97 (0.87, 1.08) P=0.538 | 1.00 (ref) | 0.90 (0.81, 1.00) P=0.049 |
| Q2 | 0.94 (0.86, 1.03) P=0.202 | 0.88 (0.79, 0.98) P=0.020 | 1.02 (0.93, 1.11) P=0.685 | 0.94 (0.85, 1.05) P=0.258 | 1.02 (0.91, 1.13) P=0.786 | 0.96 (0.87, 1.05) P=0.347 | 0.96 (0.87, 1.07) P=0.480 | 0.94 (0.86, 1.03) P=0.191 |
| Q3 | 0.97 (0.89, 1.07) P=0.591 | 0.84 (0.75, 0.94) P=0.002 | 0.95 (0.86, 1.04) P=0.281 | 1.04 (0.93, 1.15) P=0.495 | 0.98 (0.86, 1.13) P=0.795 | 0.97 (0.88, 1.06) P=0.498 | 1.01 (0.89, 1.15) P=0.845 | 0.92 (0.84, 1.01) P=0.078 |
| Q4 (most greenspace) | 0.95 (0.85, 1.06) P=0.330 | 0.96 (0.85, 1.08) P=0.461 | 0.95 (0.85, 1.06) P=0.348 | 0.96 (0.85, 1.08) P=0.492 | 0.87 (0.72, 1.06) P=0.166 | 1.02 (0.92, 1.14) P=0.681 | 0.85 (0.71, 1.02) P=0.078 | 0.91 (0.82, 1.01) P=0.066 |
|  |  |  |  |  |  |  |  |  |
| Stratum-specific HRs  (Q4 vs Q1) | 0.98 (0.86, 1.11) P=0.756 | 1.11 (0.95, 1.30) P=0.180 | 0.96 (0.84, 1.09) P=0.503 | 1.00 (0.87, 1.16) P=0.985 | 0.83 (0.66, 1.04) P=0.112 | 1.08 (0.96, 1.21) P=0.205 | 0.85 (0.68, 1.05) P=0.131 | 1.01 (0.90, 1.13) P=0.893 |
|  |  |  |  |  |  |  |  |  |
| Relative excess risk due to interaction (RERI) | 0.22 (0.10, 0.34)  P<0.001 | | 0.08 (-0.05, 0.21)  P=0.243 | | 0.18 (-0.00, 0.37)  P=0.053 | | 0.19 (-0.01, 0.33)  P=0.071 | |
| Ratio measure of effect modification on multiplicative scale^ | 1.28 (1.10, 1.46) P<0.001 | | 1.08 (0.93, 1.23) P=0.284 | | 1.21 (0.96, 1.46) P=0.074 | | 1.19 (0.95, 1.42) P=0.089 | |

* Self-reported average total household income before tax.

** 'More deprived' refers to people living in areas in the top two most deprived quintiles of the UK, based on the Townsend index.

Q = quartile

^ HR_11_ / HR_10_ x HR_01_

Note: Models are adjusted for age, ethnicity, education, employment status, urban/non-urban, assessment area, residential density, smoking status, alcohol intake, and number of years living at home address.

**3. Secondary outcomes**

**Figure S2: Hazard ratios for associations between neighbourhood characteristics and hospital admissions for breast cancer (females only) and colorectal cancer (all).**

**
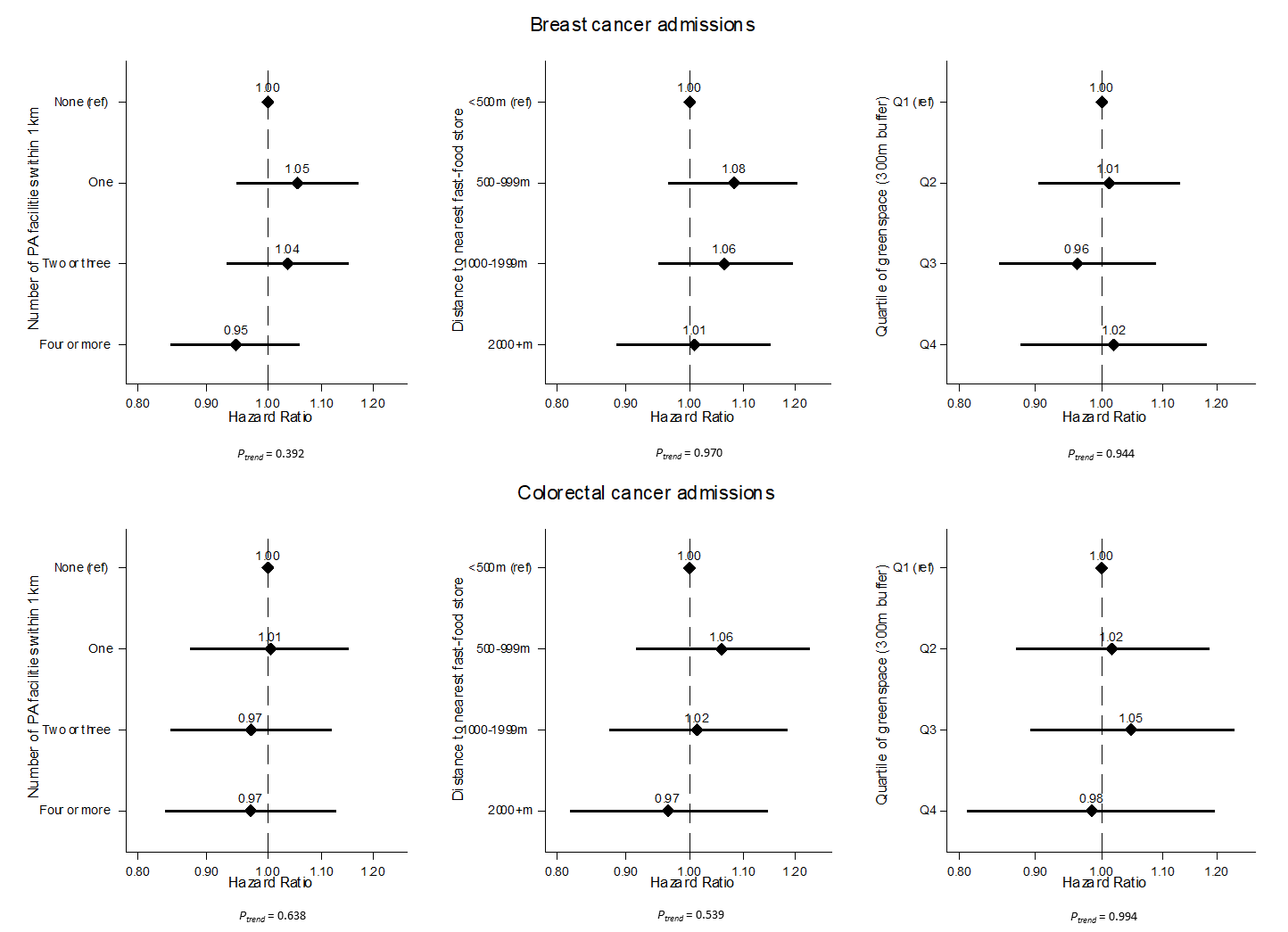
**

Note: Models are adjusted for age, sex (colorectal cancer only), ethnicity, education, household income, employment status, urban/non-urban, assessment area, residential density, smoking status, alcohol intake, and number of years living at home address.

**Table S4. Modification of the association between neighbourhood environment variables and hospital admissions due to breast cancer, by household income and area deprivation (females)**

| ***Breast cancer-related admissions*** | ***Annual household income*** | | ***Area deprivation*** | |
| --- | --- | --- | --- | --- |
|  | **Less than £31,000** | **At least £31,000** | **More deprived** | **Less deprived** |
| **Number of PA facilities** | HR (95% CI); P | HR (95% CI); P | HR (95% CI): P | HR (95% CI); P |
| None | 1.00 (ref) | 1.00 (0.87, 1.15) P=0.977 | 1.00 (ref) | 1.15 (0.95, 1.38) P=0.142 |
| One | 1.03 (0.89, 1.19) P=0.683 | 1.08 (0.92, 1.26) P=0.336 | 1.16 (0.92, 1.46) P=0.196 | 1.18 (0.97, 1.43) P=0.090 |
| 2-3 | 1.07 (0.94, 1.23) P=0.313 | 0.99 (0.85, 1.15) P=0.889 | 1.17 (0.95, 1.45) P=0.137 | 1.14 (0.94, 1.38) P=0.183 |
| 4 or more | 1.02 (0.88, 1.18) P=0.778 | 0.86 (0.73, 1.00) P=0.056 | 0.95 (0.77, 1.18) P=0.647 | 1.10 (0.91, 1.34) P=0.315 |
|  |  |  |  |  |
| Stratum-specific HRs  (4+ facilities vs 0) | 1.03 (0.88, 1.20) P=0.731 | 0.86 (0.72, 1.01) P=0.071 | 1.03 (0.81, 1.29) P=0.827 | 0.94 (0.82, 1.07) P=0.331 |
|  |  |  |  |  |
| Relative excess risk due to interaction (RERI) | -0.16 (-0.36, 0.04) P=0.109 | | 0.00 (-0.24, 0.25) P=0.975 | |
| Ratio measure of effect modification on multiplicative scale | 0.84 (0.68, 1.01) P=0.084 | | 1.01 (0.77, 1.25) P=0.941 | |
|  | **Less than £31,000** | **At least £31,000** | **More deprived** | **Less deprived** |
| **Fast-food proximity** | HR (95% CI); P | HR (95% CI); P | HR (95% CI): P | HR (95% CI); P |
| Closer than 500m | 1.00 (ref) | 0.77 (0.64, 0.93) P=0.006 | 1.00 (ref) | 0.98 (0.82, 1.16) P=0.801 |
| 500-999m | 0.95 (0.82, 1.09) P=0.447 | 1.01 (0.86, 1.17) P=0.942 | 0.96 (0.81, 1.15) P=0.690 | 1.14 (0.97, 1.33) P=0.113 |
| 1000-1999m | 0.98 (0.84, 1.13) P=0.736 | 0.94 (0.80, 1.10) P=0.449 | 1.07 (0.89, 1.29) P=0.468 | 1.07 (0.91, 1.26) P=0.405 |
| At least 2000m | 0.94 (0.80, 1.10) P=0.437 | 0.88 (0.74, 1.04) P=0.126 | 0.91 (0.72, 1.16) P=0.452 | 1.04 (0.88, 1.24) P=0.645 |
|  |  |  |  |  |
| Stratum-specific HRs  (≥2000m vs <500m) | 0.95 (0.80, 1.13) P=0.548 | 1.12 (0.91, 1.36) P=0.291 | 0.86 (0.66, 1.12) P=0.254 | 1.08 (0.92, 1.26) P=0.334 |
|  |  |  |  |  |
| Relative excess risk due to interaction (RERI) | 0.16 (-0.03, 0.35) P=0.094 | | 0.15 (-0.10, 0.41) P=0.243 | |
| Ratio measure of effect modification on multiplicative scale | 1.20 (0.93, 1.48) P=0.124 | | 1.17 (0.85, 1.49) P=0.276 | |
|  | **Less than £31,000** | **At least £31,000** | **More deprived** | **Less deprived** |
| **Greenspace** | HR (95% CI); P | HR (95% CI); P | HR (95% CI): P | HR (95% CI); P |
| Q1 (least greenspace) | 1.00 (ref) | 0.82 (0.70, 0.97) P=0.019 | 1.00 (ref) | 1.00 (0.86, 1.18) P=0.954 |
| Q2 | 0.93 (0.80, 1.07) P=0.279 | 0.95 (0.81, 1.11) P=0.525 | 1.00 (0.84, 1.18) P=0.982 | 1.06 (0.92, 1.22) P=0.432 |
| Q3 | 0.98 (0.84, 1.14) P=0.784 | 0.79 (0.67, 0.94) P=0.007 | 0.95 (0.76, 1.18) P=0.629 | 1.02 (0.88, 1.17) P=0.813 |
| Q4 (most greenspace) | 0.87 (0.73, 1.04) P=0.134 | 1.00 (0.84, 1.20) P=0.958 | 0.78 (0.57, 1.07) P=0.123 | 1.10 (0.94, 1.30) P=0.240 |
|  |  |  |  |  |
| Stratum-specific HRs  (Q4 vs Q1) | 0.84 (0.68, 1.03) P=0.097 | 1.23 (0.99, 1.53) P=0.060 | 0.69 (0.47, 0.99) P=0.044 | 1.14 (0.96, 1.35) P=0.137 |
|  |  |  |  |  |
| Relative excess risk due to interaction (RERI) | 0.31 (0.13, 0.49) P=0.001 | | 0.32 (0.04, 0.59) P=0.024 | |
| Ratio measure of effect modification on multiplicative scale | 1.40 (1.10, 1.69) P=0.002 | | 1.40 (0.94, 1.87) P=0.055 | |

Note: Models are adjusted for age, ethnicity, education, employment status, urban/non-urban, assessment area, residential density, smoking status, alcohol intake, and number of years living at home address.

**Table S5. Association between neighbourhood characteristics and breast cancer-related hospital admissions, stratified by household income and area deprivation in combination (females)**

| ***Breast cancer-related admissions*** | ***Combined household income and area deprivation*** | | | |
| --- | --- | --- | --- | --- |
|  | **Less than £31,000 & more deprived** | **At least £31,000 & more deprived** | **Less than £31,000 & less deprived** | **At least £31,000 & less deprived** |
|  | HR (95% CI); P | HR (95% CI); P | HR (95% CI); P | HR (95% CI); P |
| **Number of PA facilities** |  |  |  |  |
| None | 1.00 (ref) | 1.00 (ref) | 1.00 (ref) | 1.00 (ref) |
| One | 1.22 (0.92, 1.61) P=0.165 | 1.13 (0.74, 1.73) P=0.581 | 0.97 (0.81, 1.15) P=0.698 | 1.08 (0.91, 1.27) P=0.385 |
| 2-3 | 1.29 (0.99, 1.68) P=0.055 | 1.07 (0.71, 1.60) P=0.747 | 0.97 (0.82, 1.16) P=0.757 | 0.98 (0.82, 1.16) P=0.794 |
| 4 or more | 1.11 (0.84, 1.48) P=0.450 | 0.85 (0.56, 1.29) P=0.444 | 1.01 (0.84, 1.22) P=0.912 | 0.87 (0.72, 1.05) P=0.158 |
| P*_trend_* | 0.509 | 0.273 | 0.968 | 0.157 |
| **Fast-food proximity** |  |  |  |  |
| Closer than 500m | 1.00 (ref) | 1.00 (ref) | 1.00 (ref) | 1.00 (ref) |
| 500-999m | 0.87 (0.70, 1.09) P=0.234 | 1.11 (0.82, 1.51) P=0.511 | 1.00 (0.82, 1.22) P=0.985 | 1.40 (1.12, 1.74) P=0.003 |
| 1000-1999m | 1.00 (0.78, 1.27) P=0.977 | 1.05 (0.73, 1.50) P=0.799 | 0.99 (0.81, 1.21) P=0.955 | 1.27 (1.02, 1.59) P=0.031 |
| At least 2000m | 0.85 (0.62, 1.17) P=0.321 | 0.87 (0.53, 1.43) P=0.575 | 0.99 (0.80, 1.22) P=0.902 | 1.22 (0.96, 1.54) P=0.104 |
| P*_trend_* | 0.552 | 0.770 | 0.879 | 0.565 |
| **Greenspace** |  |  |  |  |
| Q1 (least greenspace) | 1.00 (ref) | 1.00 (ref) | 1.00 (ref) | 1.00 (ref) |
| Q2 | 0.93 (0.75, 1.15) P=0.489 | 0.99 (0.72, 1.37) P=0.973 | 0.95 (0.77, 1.17) P=0.636 | 1.13 (0.91, 1.41) P=0.252 |
| Q3 | 0.94 (0.72, 1.23) P=0.639 | 0.64 (0.40, 1.03) P=0.068 | 1.05 (0.85, 1.30) P=0.629 | 0.99 (0.79, 1.23) P=0.898 |
| Q4 (most greenspace) | 0.61 (0.38, 0.97) P=0.036 | 0.80 (0.43, 1.50) P=0.486 | 0.92 (0.72, 1.19) P=0.533 | 1.35 (1.05, 1.73) P=0.017 |
| P*_trend_* | 0.124 | 0.159 | 0.858 | 0.052 |

Note: Models are adjusted for age, ethnicity, education, employment status, urban/non-urban, assessment area, residential density, smoking status, alcohol intake, and number of years living at home address.

**Table S6. Modification of the association between neighbourhood environment variables and hospital admissions due to colorectal cancer, by household income and area deprivation**

| ***Colorectal cancer-related admissions*** | ***Annual household income*** | | ***Area deprivation*** | |
| --- | --- | --- | --- | --- |
|  | **Less than £31,000** | **At least £31,000** | **More deprived** | **Less deprived** |
| **Number of PA facilities** | HR (95% CI); P | HR (95% CI); P | HR (95% CI): P | HR (95% CI); P |
| None | 1.00 (ref) | 0.85 (0.71, 1.02) P=0.077 | 1.00 (ref) | 1.31 (1.02, 1.70) P=0.035 |
| One | 0.95 (0.80, 1.13) P=0.566 | 0.93 (0.75, 1.14) P=0.477 | 1.34 (0.98, 1.83) P=0.068 | 1.24 (0.95, 1.62) P=0.111 |
| 2-3 | 0.92 (0.77, 1.10) P=0.352 | 0.89 (0.73, 1.09) P=0.274 | 1.18 (0.88, 1.59) P=0.268 | 1.23 (0.95, 1.60) P=0.118 |
| 4 or more | 0.92 (0.77, 1.10) P=0.381 | 0.89 (0.72, 1.09) P=0.243 | 1.26 (0.95, 1.67) P=0.112 | 1.18 (0.91, 1.55) P=0.216 |
|  |  |  |  |  |
| Stratum-specific HRs  (4+ facilities vs 0) | 0.87 (0.72, 1.06) P=0.169 | 1.13 (0.89, 1.42) P=0.313 | 1.33 (0.97, 1.81) P=0.073 | 0.87 (0.73, 1.04) P=0.123 |
|  |  |  |  |  |
| Relative excess risk due to interaction (RERI) | 0.12 (-0.11, 0.35) P=0.325 | | -0.39 (-0.81, 0.03) P=0.071 | |
| Ratio measure of effect modification on multiplicative scale | 1.13 (0.84, 1.43) P=0.374 | | 0.72 (0.49, 0.94) P=0.048 | |
|  | **Less than £31,000** | **At least £31,000** | **More deprived** | **Less deprived** |
| **Fast-food proximity** | HR (95% CI); P | HR (95% CI); P | HR (95% CI): P | HR (95% CI); P |
| Closer than 500m | 1.00 (ref) | 0.91 (0.71, 1.16) P=0.431 | 1.00 (ref) | 0.99 (0.78, 1.25) P=0.924 |
| 500-999m | 1.12 (0.93, 1.34) P=0.225 | 0.88 (0.71, 1.09) P=0.248 | 0.94 (0.74, 1.19) P=0.582 | 1.12 (0.91, 1.37) P=0.296 |
| 1000-1999m | 1.03 (0.85, 1.25) P=0.744 | 0.91 (0.73, 1.13) P=0.405 | 1.12 (0.87, 1.43) P=0.368 | 0.99 (0.80, 1.22) P=0.914 |
| At least 2000m | 0.87 (0.71, 1.07) P=0.195 | 1.00 (0.80, 1.25) P=0.984 | 0.87 (0.63, 1.21) P=0.414 | 0.99 (0.79, 1.24) P=0.917 |
|  |  |  |  |  |
| Stratum-specific HRs  (≥2000m vs <500m) | 0.89 (0.71, 1.11) P=0.290 | 1.06 (0.81, 1.40) P=0.650 | 0.87 (0.61, 1.24) P=0.440 | 1.00 (0.82, 1.23) P=0.967 |
|  |  |  |  |  |
| Relative excess risk due to interaction (RERI) | 0.22 (-0.05, 0.49) P=0.107 | | 0.12 (-0.21, 0.46) P=0.462 | |
| Ratio measure of effect modification on multiplicative scale | 1.26 (0.88, 1.64) P=0.146 | | 1.14 (0.72, 1.56) P=0.517 | |
|  | **Less than £31,000** | **At least £31,000** | **More deprived** | **Less deprived** |
| **Greenspace** | HR (95% CI); P | HR (95% CI); P | HR (95% CI); P | HR (95% CI); P |
| Q1 (least greenspace) | 1.00 (ref) | 0.87 (0.69, 1.09) P=0.214 | 1.00 (ref) | 0.96 (0.77, 1.20) P=0.745 |
| Q2 | 1.02 (0.85, 1.23) P=0.805 | 0.88 (0.70, 1.10) P=0.248 | 0.98 (0.78, 1.23) P=0.844 | 1.02 (0.85, 1.23) P=0.825 |
| Q3 | 0.99 (0.81, 1.20) P=0.911 | 1.00 (0.81, 1.24) P=0.991 | 1.04 (0.79, 1.38) P=0.760 | 1.05 (0.87, 1.26) P=0.643 |
| Q4 (most greenspace) | 0.96 (0.77, 1.20) P=0.717 | 0.89 (0.70, 1.14) P=0.367 | 0.77 (0.51, 1.15) P=0.203 | 1.01 (0.81, 1.25) P=0.962 |
|  |  |  |  |  |
| Stratum-specific HRs  (Q4 vs Q1) | 1.04 (0.80, 1.34) P=0.779 | 0.92 (0.68, 1.26) P=0.619 | 0.69 (0.43, 1.12) P=0.135 | 1.08 (0.86, 1.37) P=0.494 |
|  |  |  |  |  |
| Relative excess risk due to interaction (RERI) | 0.07 (-0.19, 0.33) P=0.601 | | 0.27 (-0.08, 0.63) P=0.133 | |
| Ratio measure of effect modification on multiplicative scale | 1.08 (0.77, 1.38) P=0.618 | | 1.36 (0.76, 1.95) P=0.202 | |

Note: Models are adjusted for age, sex, ethnicity, education, employment status, urban/non-urban, assessment area, residential density, smoking status, alcohol intake, and number of years living at home address.

**Table S7. Association between neighbourhood characteristics and colorectal cancer-related hospital admissions, stratified by household income and area deprivation in combination**

| ***Colorectal cancer-related admissions*** | ***Combined household income and area deprivation*** | | | |
| --- | --- | --- | --- | --- |
|  | **Less than £31,000 & more deprived** | **At least £31,000 & more deprived** | **Less than £31,000 & less deprived** | **At least £31,000 & less deprived** |
|  | HR (95% CI); P | HR (95% CI); P | HR (95% CI); P | HR (95% CI); P |
| **Number of PA facilities** |  |  |  |  |
| None | 1.00 (ref) | 1.00 (ref) | 1.00 (ref) | 1.00 (ref) |
| One | 1.37 (0.95, 1.98) P=0.090 | 1.33 (0.71, 2.48) P=0.373 | 0.83 (0.67, 1.02) P=0.077 | 1.10 (0.87, 1.39) P=0.421 |
| 2-3 | 1.28 (0.90, 1.83) P=0.173 | 1.11 (0.61, 2.03) P=0.724 | 0.80 (0.65, 0.99) P=0.043 | 1.12 (0.88, 1.42) P=0.367 |
| 4 or more | 1.38 (0.96, 1.99) P=0.084 | 1.21 (0.67, 2.19) P=0.533 | 0.72 (0.56, 0.91) P=0.006 | 1.11 (0.86, 1.44) P=0.414 |
| *P_trend_* | 0.154 | 0.733 | 0.005 | 0.377 |
| **Fast-food proximity** |  |  |  |  |
| Closer than 500m | 1.00 (ref) | 1.00 (ref) | 1.00 (ref) | 1.00 (ref) |
| 500-999m | 1.01 (0.76, 1.34) P=0.949 | 0.83 (0.53, 1.31) P=0.427 | 1.22 (0.96, 1.56) P=0.111 | 1.00 (0.74, 1.35) P=0.999 |
| 1000-1999m | 1.10 (0.81, 1.50) P=0.549 | 1.25 (0.77, 2.03) P=0.359 | 1.05 (0.81, 1.35) P=0.713 | 0.93 (0.69, 1.26) P=0.638 |
| At least 2000m | 0.84 (0.55, 1.27) P=0.400 | 0.99 (0.51, 1.92) P=0.984 | 0.92 (0.69, 1.21) P=0.539 | 1.09 (0.80, 1.49) P=0.592 |
| *P_trend_* | 0.749 | 0.615 | 0.181 | 0.587 |
| **Greenspace** |  |  |  |  |
| Q1 (least greenspace) | 1.00 (ref) | 1.00 (ref) | 1.00 (ref) | 1.00 (ref) |
| Q2 | 1.03 (0.79, 1.36) P=0.807 | 0.68 (0.41, 1.12) P=0.132 | 1.08 (0.82, 1.40) P=0.593 | 1.07 (0.77, 1.47) P=0.697 |
| Q3 | 1.05 (0.74, 1.48) P=0.797 | 0.85 (0.47, 1.53) P=0.591 | 1.06 (0.81, 1.40) P=0.659 | 1.17 (0.85, 1.61) P=0.330 |
| Q4 (most greenspace) | 0.68 (0.37, 1.23) P=0.203 | 0.67 (0.28, 1.59) P=0.366 | 1.14 (0.83, 1.56) P=0.42 | 1.05 (0.73, 1.50) P=0.802 |
| *P_trend_* | 0.599 | 0.306 | 0.494 | 0.764 |

Note: Models are adjusted for age, sex, ethnicity, education, employment status, urban/non-urban, assessment area, residential density, smoking status, alcohol intake, and number of years living at home address.

**4. Sensitivity analyses: Restricting follow-up time to January 2012 onwards**

**Table S8. Hospital admissions by household income and area deprivation (follow-up time restricted to January 2012 onwards)**

|  |  | |
| --- | --- | --- |
|  | N | Number of cancer admissions (%) |
| Total | 320812 | 8168 (2.5) |
| Household income (annual pre-tax) |  |  |
| <£31,000 | 151885 | 4728 (3.1) |
| £31,000 or more | 168927 | 3440 (2.0) |
| Area deprivation |  |  |
| More deprived | 94194 | 2419 (2.6) |
| Less deprived | 226618 | 5749 (2.5) |

**Table S9. Modification of the associations between neighbourhood environment variables and hospital admissions due to cancer, by household income and area deprivation (follow-up time restricted to January 2012 onwards)**

| ***All cancer-related admissions*** | ***Annual household income*** | | ***Area deprivation*** | |
| --- | --- | --- | --- | --- |
|  | **Less than £31,000** | **At least £31,000** | **More deprived** | **Less deprived** |
| **Number of PA facilities** | HR (95% CI) | HR (95% CI) | HR (95% CI) | HR (95% CI) |
| None | 1.00 (ref) | 0.98 (0.90, 1.07) P=0.669 | 1.00 (ref) | 1.01 (0.91, 1.12) P=0.846 |
| One | 0.96 (0.88, 1.04) P=0.317 | 0.94 (0.85, 1.04) P=0.212 | 1.07 (0.94, 1.22) P=0.276 | 0.93 (0.84, 1.04) P=0.220 |
| 2-3 | 0.99 (0.91, 1.07) P=0.781 | 0.90 (0.82, 0.98) P=0.022 | 1.00 (0.89, 1.13) P=0.971 | 0.96 (0.86, 1.07) P=0.457 |
| 4 or more | 0.96 (0.88, 1.04) P=0.328 | 0.89 (0.81, 0.98) P=0.014 | 0.96 (0.86, 1.08) P=0.538 | 0.95 (0.85, 1.06) P=0.385 |
|  |  |  |  |  |
| Stratum-specific HRs (4+ facilities vs 0) | 0.93 (0.86, 1.02) P=0.131 | 0.94 (0.84, 1.04) P=0.218 | 0.94 (0.83, 1.07) P=0.346 | 0.95 (0.87, 1.03) P=0.181 |
|  |  |  |  |  |
| Relative excess risk due to interaction (RERI) | -0.05 (-0.17, 0.06) P=0.362 | | -0.02 (-0.16, 0.11) P=0.751 | |
| Ratio measure of effect modification on multiplicative scale | 0.94 (0.83, 1.05) P=0.307 | | 0.98 (0.85, 1.11) P=0.762 | |
| **Fast-food proximity** | HR (95% CI) | HR (95% CI) | HR (95% CI) | HR (95% CI) |
| Closer than 500m | 1.00 (ref) | 0.86 (0.77, 0.96) P=0.008 | 1.00 (ref) | 0.93 (0.84, 1.03) P=0.188 |
| 500-999m | 0.92 (0.85, 1.00) P=0.053 | 0.91 (0.83, 1.01) P=0.065 | 0.91 (0.82, 1.01) P=0.077 | 0.94 (0.86, 1.03) P=0.170 |
| 1000-1999m | 0.99 (0.91, 1.07) P=0.760 | 0.91 (0.83, 1.01) P=0.065 | 1.05 (0.95, 1.17) P=0.330 | 0.93 (0.85, 1.02) P=0.133 |
| At least 2000m | 0.94 (0.85, 1.03) P=0.163 | 0.93 (0.84, 1.02) P=0.132 | 0.91 (0.79, 1.05) P=0.184 | 0.94 (0.85, 1.04) P=0.236 |
|  |  |  |  |  |
| Stratum-specific HRs (≥2000m vs <500m) | 0.97 (0.88, 1.07) P=0.576 | 1.01 (0.89. 1.14) P=0.860 | 0.93 (0.80, 1.08) P=0.315 | 1.00 (0.91, 1.10) P=0.946 |
|  |  |  |  |  |
| Relative excess risk due to interaction (RERI) | 0.12 (0.00, 0.25) P=0.042 | | 0.10 (-0.05, 0.24) P=0.196 | |
| Ratio measure of effect modification on multiplicative scale | 1.14 (0.99, 1.30) P=0.059 | | 1.11 (0.93, 1.28) P=0.202 | |
| **Greenspace** | HR (95% CI) | HR (95% CI) | HR (95% CI) | HR (95% CI) |
| Q1 (least greenspace) | 1.00 (ref) | 0.87 (0.79, 0.96) P=0.006 | 1.00 (ref) | 0.94 (0.85, 1.04) P=0.218 |
| Q2 | 0.98 (0.91, 1.07) P=0.668 | 0.87 (0.79, 0.97) P=0.008 | 0.98 (0.89, 1.09) P=0.740 | 0.93 (0.86, 1.01) P=0.106 |
| Q3 | 0.93 (0.85, 1.02) P=0.109 | 0.94 (0.85, 1.04) P=0.244 | 0.96 (0.85, 1.09) P=0.567 | 0.93 (0.86, 1.01) P=0.105 |
| Q4 (most greenspace) | 0.93 (0.84, 1.04) P=0.199 | 0.94 (0.85, 1.05) P=0.300 | 0.86 (0.72, 1.02) P=0.081 | 0.95 (0.86, 1.05) P=0.306 |
|  |  |  |  |  |
| Stratum-specific HRs (Q4 vs Q1) | 0.97 (0.86, 1.09) P=0.574 | 1.02 (0.89, 1.17) P=0.768 | 0.87 (0.70, 1.06) P=0.166 | 1.01 (0.91, 1.23) P=0.789 |
|  |  |  |  |  |
| Relative excess risk due to interaction (RERI) | 0.14 (0.02, 0.25) P=0.019 | | 0.15 (-0.01, 0.31) P=0.072 | |
| Ratio measure of effect modification on multiplicative scale | 1.16 (1.01, 1.31) P=0.025 | | 1.18 (0.96, 1.39) P=0.079 | |

Note: Models are adjusted for age, sex, ethnicity, education, employment status, urban/non-urban, assessment area, residential density, smoking status, alcohol intake, and number of years living at home address.

**Table S10. Association between neighbourhood characteristics and cancer-related hospital admissions, stratified by household income and area deprivation in combination (follow-up time restricted to January 2012 onwards)**

| ***All cancer-related admissions*** | ***Combined household income and area deprivation*** | | | |
| --- | --- | --- | --- | --- |
|  | **Less than £31,000 & more deprived** | **At least £31,000 & more deprived** | **Less than £31,000 & less deprived** | **At least £31,000 & less deprived** |
|  | HR (95% CI) | HR (95% CI) | HR (95% CI) | HR (95% CI) |
| **Number of PA facilities** |  |  |  |  |
| None | 1.00 (ref) | 1.00 (ref) | 1.00 (ref) | 1.00 (ref) |
| One | 1.01 (0.87, 1.18) P=0.876 | 1.18 (0.91, 1.53) P=0.203 | 0.92 (0.83, 1.02) P=0.106 | 0.94 (0.84, 1.04) P=0.232 |
| 2-3 | 1.00 (0.86, 1.15) P=0.964 | 0.95 (0.74, 1.22) P=0.681 | 0.96 (0.87, 1.07) P=0.488 | 0.94 (0.84, 1.05) P=0.263 |
| 4 or more | 0.94 (0.80, 1.09) P=0.390 | 0.95 (0.75, 1.22) P=0.702 | 0.93 (0.83, 1.04) P=0.216 | 0.95 (0.85, 1.07) P=0.425 |
| *P_trend_* | 0.345 | 0.278 | 0.292 | 0.373 |
|  | HR (95% CI) | HR (95% CI) | HR (95% CI) | HR (95% CI) |
| **Fast-food proximity** |  |  |  |  |
| Closer than 500m | 1.00 (ref) | 1.00 (ref) | 1.00 (ref) | 1.00 (ref) |
| 500-999m | 0.93 (0.82, 1.05) P=0.221 | 0.90 (0.74, 1.08) P=0.267 | 0.94 (0.84, 1.06) P=0.308 | 1.10 (0.96, 1.27) P=0.153 |
| 1000-1999m | 1.05 (0.92, 1.20) P=0.504 | 1.09 (0.88, 1.34) P=0.432 | 1.00 (0.89, 1.12) P=0.995 | 1.01 (0.88, 1.16) P=0.862 |
| At least 2000m | 0.89 (0.75, 1.06) P=0.206 | 1.03 (0.78, 1.36) P=0.840 | 1.00 (0.88, 1.13) P=0.968 | 1.03 (0.90, 1.20) P=0.643 |
| *P_trend_* | 0.650 | 0.525 | 0.660 | 0.785 |
|  | HR (95% CI) | HR (95% CI) | HR (95% CI) | HR (95% CI) |
| **Greenspace** |  |  |  |  |
| Q1 (least greenspace) | 1.00 (ref) | 1.00 (ref) | 1.00 (ref) | 1.00 (ref) |
| Q2 | 1.00 (0.89, 1.13) P=0.965 | 0.94 (0.77, 1.15) P=0.528 | 1.03 (0.91, 1.16) P=0.675 | 0.97 (0.84, 1.11) P=0.630 |
| Q3 | 0.92 (0.79, 1.08) P=0.310 | 1.08 (0.84, 1.39) P=0.556 | 1.00 (0.88, 1.14) P=0.987 | 1.02 (0.88, 1.17) P=0.805 |
| Q4 (most greenspace) | 0.76 (0.59, 0.97) P=0.031 | 1.17 (0.82, 1.67) P=0.388 | 1.04 (0.90, 1.21) P=0.593 | 1.02 (0.86, 1.19) P=0.850 |
| *P_trend_* | 0.071 | 0.453 | 0.762 | 0.591 |

Note: Models are adjusted for age, sex, ethnicity, education, employment status, urban/non-urban, assessment area, residential density, smoking status, alcohol intake, and number of years living at home address.

**5. Sensitivity analyses: Models additionally adjusted for baseline BMI, hypertension and medications for hypertension and high cholesterol**

**Table S11. Modification of the association between neighbourhood variables and admissions, by household income and area deprivation (with additional adjustment)**

| ***All cancer-related admissions*** | ***Annual household income*** | | ***Area deprivation*** | |
| --- | --- | --- | --- | --- |
|  | **Less than £31,000** | **At least £31,000** | **More deprived** | **Less deprived** |
| **Number of PA facilities** | HR (95% CI) | HR (95% CI) | HR (95% CI) | HR (95% CI) |
| None | 1.00 (ref) | 0.96 (0.90, 1.02) P=0.224 | 1.00 (ref) | 1.03 (0.95, 1.11) P=0.530 |
| One | 0.98 (0.92, 1.05) P=0.594 | 0.99 (0.92, 1.06) P=0.728 | 1.08 (0.97, 1.20) P=0.145 | 1.01 (0.93, 1.10) P=0.854 |
| 2-3 | 1.00 (0.94, 1.06) P=0.939 | 0.94 (0.88, 1.01) P=0.094 | 1.06 (0.96, 1.16) P=0.238 | 1.00 (0.92, 1.09) P=0.979 |
| 4 or more | 0.99 (0.93, 1.05) P=0.695 | 0.90 (0.83, 0.97) P=0.004 | 1.01 (0.92, 1.11) P=0.775 | 0.98 (0.90, 1.07) P=0.722 |
|  |  |  |  |  |
| Stratum-specific HRs (4+ facilities vs 0) | 0.97 (0.91, 1.04) P=0.397 | 0.96 (0.88, 1.04) P=0.277 | 1.02 (0.93, 1.13) P=0.643 | 0.96 (0.90, 1.02) P=0.152 |
| Relative excess risk due to interaction (RERI) | -0.05 (-0.14, 0.04) P=0.261 | | -0.06 (-0.16, 0.05) P=0.316 | |
| Ratio measure of effect modification on multiplicative scale | 0.95 (0.86, 1.03) P=0.268 | | 0.95 (0.85, 1.05) P=0.347 | |
| **Fast-food proximity** | HR (95% CI) | HR (95% CI) | HR (95% CI) | HR (95% CI) |
| Closer than 500m | 1.00 (ref) | 0.89 (0.82, 0.97) P=0.007 | 1.00 (ref) | 0.92 (0.85, 0.99) P=0.028 |
| 500-999m | 0.93 (0.88, 1.00) P=0.035 | 0.93 (0.86, 1.00) P=0.046 | 0.90 (0.83, 0.97) P=0.006 | 0.94 (0.87, 1.01) P=0.076 |
| 1000-1999m | 0.97 (0.91, 1.04) P=0.392 | 0.91 (0.84, 0.98) P=0.010 | 0.98 (0.90, 1.07) P=0.685 | 0.92 (0.85, 0.99) P=0.019 |
| At least 2000m | 0.94 (0.87, 1.01) P=0.076 | 0.91 (0.84, 0.98) P=0.019 | 0.92 (0.83, 1.03) P=0.137 | 0.91 (0.85, 0.99) P=0.020 |
|  |  |  |  |  |
| Stratum-specific HRs  (≥2000m vs <500m) | 0.97 (0.90, 1.04) P=0.369 | 0.97 (0.88, 1.07) P=0.547 | 0.92 (0.82, 1.03) P=0.150 | 1.00 (0.93, 1.07) P=0.927 |
| Relative excess risk due to interaction (RERI) | 0.08 (-0.01, 0.18) P=0.090 | | 0.07 (-0.04, 0.19) P=0.200 | |
| Ratio measure of effect modification on multiplicative scale | 1.09 (0.98, 1.20) P=0.095 | | 1.08 (0.95, 1.21) P=0.214 | |
| **Greenspace** | HR (95% CI) | HR (95% CI) | HR (95% CI) | HR (95% CI) |
| Q1 (least greenspace) | 1.00 (ref) | 0.86 (0.80, 0.93) P=0.000 | 1.00 (ref) | 0.94 (0.87, 1.02) P=0.118 |
| Q2 | 0.98 (0.92, 1.04) P=0.464 | 0.91 (0.84, 0.98) P=0.011 | 0.99 (0.92, 1.07) P=0.783 | 0.95 (0.89, 1.02) P=0.135 |
| Q3 | 0.96 (0.89, 1.02) P=0.192 | 0.94 (0.87, 1.02) P=0.122 | 1.00 (0.91, 1.10) P=0.985 | 0.95 (0.89, 1.01) P=0.122 |
| Q4 (most greenspace) | 0.94 (0.87, 1.02) P=0.132 | 0.96 (0.88, 1.04) P=0.285 | 0.85 (0.75, 0.97) P=0.019 | 0.97 (0.90, 1.04) P=0.390 |
|  |  |  |  |  |
| Stratum-specific HRs (Q4 vs Q1) | 0.96 (0.88, 1.05) P=0.392 | 1.05 (0.95, 1.17) P=0.339 | 0.83 (0.71, 0.97) P=0.021 | 1.04 (0.95, 1.12) P=0.393 |
| Relative excess risk due to interaction (RERI) | 0.15 (0.06, 0.24) P=0.001 | | 0.17 (0.05, 0.30) P=0.008 | |
| Ratio measure of effect modification on multiplicative scale | 1.18 (1.06, 1.29) P=0.001 | | 1.20 (1.03, 1.38) P=0.014 | |

Note: Models adjusted for age, sex, ethnicity, education, employment status, urban/non-urban, assessment area, residential density, smoking, alcohol intake, years at home address, baseline BMI, hypertension and medications for hypertension and high cholesterol.

**Table S12. Association between neighbourhood characteristics and cancer-related hospital admissions, stratified by household income and area deprivation in combination (adjusted for additional risk factors)**

| ***All cancer-related admissions*** | ***Combined household income and area deprivation*** | | | |
| --- | --- | --- | --- | --- |
|  | **Less than £31,000 & more deprived** | **At least £31,000 & more deprived** | **Less than £31,000 & less deprived** | **At least £31,000 & less deprived** |
|  | HR (95% CI) | HR (95% CI) | HR (95% CI) | HR (95% CI) |
| **Number of PA facilities** |  |  |  |  |
| None | 1.00 (ref) | 1.00 (ref) | 1.00 (ref) | 1.00 (ref) |
| One | 1.06 (0.94, 1.19) P=0.377 | 1.13 (0.92, 1.39) P=0.246 | 0.95 (0.88, 1.02) P=0.182 | 1.02 (0.94, 1.11) P=0.589 |
| 2-3 | 1.07 (0.96, 1.20) P=0.228 | 1.04 (0.85, 1.26) P=0.712 | 0.96 (0.88, 1.03) P=0.252 | 0.99 (0.91, 1.07) P=0.786 |
| 4 or more | 1.05 (0.93, 1.18) P=0.403 | 0.98 (0.80, 1.19) P=0.804 | 0.94 (0.86, 1.02) P=0.156 | 0.97 (0.88, 1.06) P=0.449 |
| *P_trend_* | 0.434 | 0.417 | 0.155 | 0.417 |
|  | HR (95% CI) | HR (95% CI) | HR (95% CI) | HR (95% CI) |
| **Fast-food proximity** |  |  |  |  |
| Closer than 500m | 1.00 (ref) | 1.00 (ref) | 1.00 (ref) | 1.00 (ref) |
| 500-999m | 0.88 (0.80, 0.97) P=0.009 | 0.93 (0.80, 1.08) P=0.352 | 1.00 (0.91, 1.09) P=0.996 | 1.06 (0.96, 1.18) P=0.249 |
| 1000-1999m | 0.96 (0.86, 1.06) P=0.418 | 1.01 (0.86, 1.20) P=0.880 | 1.03 (0.94, 1.12) P=0.558 | 0.98 (0.89, 1.09) P=0.742 |
| At least 2000m | 0.87 (0.76, 1.00) P=0.046 | 1.06 (0.85, 1.32) P=0.597 | 1.02 (0.93, 1.12) P=0.681 | 0.98 (0.88, 1.09) P=0.702 |
| *P_trend_* | 0.112 | 0.609 | 0.549 | 0.238 |
|  | HR (95% CI) | HR (95% CI) | HR (95% CI) | HR (95% CI) |
| **Greenspace** |  |  |  |  |
| Q1 (least greenspace) | 1.00 (ref) | 1.00 (ref) | 1.00 (ref) | 1.00 (ref) |
| Q2 | 0.99 (0.90, 1.08) P=0.778 | 0.96 (0.82, 1.13) P=0.641 | 1.01 (0.92, 1.11) P=0.859 | 1.03 (0.92, 1.15) P=0.617 |
| Q3 | 0.96 (0.85, 1.08) P=0.484 | 1.04 (0.85, 1.27) P=0.729 | 1.00 (0.91, 1.10) P=0.983 | 1.05 (0.94, 1.18) P=0.365 |
| Q4 (most greenspace) | 0.75 (0.62, 0.91) P=0.003 | 1.06 (0.79, 1.41) P=0.702 | 1.02 (0.91, 1.15) P=0.687 | 1.08 (0.95, 1.22) P=0.234 |
| *P_trend_* | 0.035 | 0.727 | 0.765 | 0.205 |

Note: Models adjusted for age, sex, ethnicity, education, employment status, urban/non-urban, assessment area, residential density, smoking, alcohol intake, years at home address, baseline BMI, hypertension and medications for hypertension and high cholesterol.

**6. Examination of proportional hazards assumption (primary models)**


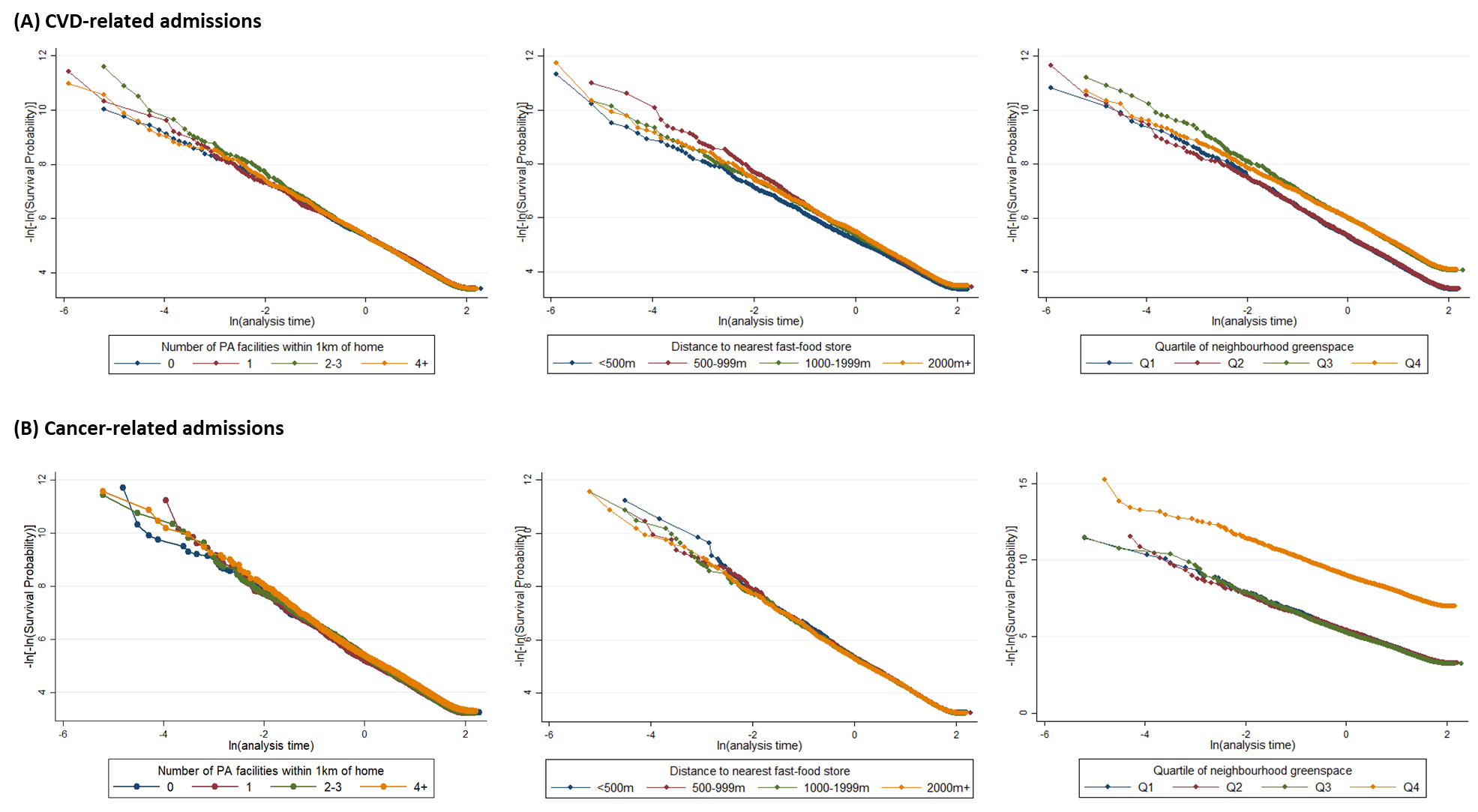


**Figure S3. Log-log plots (adjusted for all covariates) for graphical examination of proportional hazards assumption**
